# A Global Analysis of Photoreceptor-Mediated Transcriptional Changes Reveals the Intricate Relationship Between Central Metabolism and DNA Repair in the Filamentous Fungus *Trichoderma atroviride*

**DOI:** 10.3389/fmicb.2021.724676

**Published:** 2021-09-08

**Authors:** Enrique Pola-Sánchez, José Manuel Villalobos-Escobedo, Nohemí Carreras-Villaseñor, Pedro Martínez-Hernández, Emma Beatriz Beltrán-Hernández, Edgardo Ulises Esquivel-Naranjo, Alfredo Herrera-Estrella

**Affiliations:** ^1^Laboratorio Nacional de Genómica para la Biodiversidad-Unidad de Genómica Avanzada, Centro de Investigación y de Estudios Avanzados del IPN, Irapuato, Mexico; ^2^Red de Estudios Moleculares Avanzados, Instituto de Ecología A.C, Xalapa, Mexico; ^3^Laboratorio de Microbiología Molecular, Unidad de Microbiología Básica y Aplicada, Facultad de Ciencias Naturales, Universidad Autónoma de Querétaro, Querétaro, Mexico

**Keywords:** RNA-seq, photoreceptors, DNA repair, metabolism, blue light, photoreactivation

## Abstract

Light provides critical information for the behavior and development of basically all organisms. Filamentous fungi sense blue light, mainly, through a unique transcription factor complex that activates its targets in a light-dependent manner. In *Trichoderma atroviride*, the BLR-1 and BLR-2 proteins constitute this complex, which triggers the light-dependent formation of asexual reproduction structures (conidia). We generated an ENVOY photoreceptor mutant and performed RNA-seq analyses in the mutants of this gene and in those of the BLR-1, CRY-1 and CRY-DASH photoreceptors in response to a pulse of low intensity blue light. Like in other filamentous fungi BLR-1 appears to play a central role in the regulation of blue-light responses. Phenotypic characterization of the Δ*env*-1 mutant showed that ENVOY functions as a growth and conidiation checkpoint, preventing exacerbated light responses. Similarly, we observed that CRY-1 and CRY-DASH contribute to the typical light-induced conidiation response. In the Δ*env*-1 mutant, we observed, at the transcriptomic level, a general induction of DNA metabolic processes and strong repression of central metabolism. An analysis of the expression level of DNA repair genes showed that they increase their expression in the absence of *env*-1. Consistently, photoreactivation experiments showed that Δ*env*-1 had increased DNA repair capacity. Our results indicate that light perception in *T. atroviride* is far more complex than originally thought.

## Introduction

Light is an environmental clue for the behavior, development, and physiology of living beings, accordingly, they have developed sophisticated mechanisms to perceive and respond to the quality, quantity, and direction of this stimulus. Light perception is mediated by chromophore binding proteins that act as receptors for its transduction (Linden et al., 1997; Fankhauser, 2001; Ma et al., 2001; Corrochano, 2019). The energy of light is perceived by the photoreceptors, leading to the adoption of an active state of the protein (Chen et al., 2004; Ko et al., 2007). In the active state, the photoreceptor transduces the light signal to effectors that regulate the expression of genes that trigger physiological responses in the cell (Froehlich et al., 2002; Chen et al., 2004; Ko et al., 2007). Depending on the type of photoreceptor, differences in wavelength, intensity, direction, and duration of light can be perceived and responded to Terzaghi and Cashmore (1995) and Falciatore and Bowler (2005).

Fungi use light as a source of information about the environment that surrounds them. Due to their relative simplicity, filamentous fungi have been used as models to understand the mechanisms of light perception. Among the responses mediated by light in filamentous fungi are conidiation, phototropism, sexual development, entrainment of the circadian rhythm, photomorphogenesis and secondary metabolism (Herrera-Estrella and Horwitz, 2007; Rodríguez-Romero et al., 2010). Although it is known that there are responses regulated by green, red and ultraviolet light, blue light regulates most responses in fungi (Herrera-Estrella and Horwitz, 2007).

For decades, the filamentous fungus *Neurospora crassa* has been the workhorse for studies on light perception and, where the first gene encoding a fungal photoreceptor, *wc-*1, was identified. The *wc*-1 gene codes for the White Collar-1 (WC-1) protein, which has three PAS-like domains (Per-Arnt-Sim), one of which belongs to a subfamily of domains specialized in perceiving Light, Oxygen or Voltage signals (LOV). In the LOV domain, FAD is covalently bound, which acts as a chromophore allowing WC-1 to function as a photoreceptor of blue light (He et al., 2002; Froehlich et al., 2002). The *wc*-2 gene codes for the White Collar-2 (WC-2) protein, which, like WC-1, has a nuclear localization signal, but has only one PAS domain. The PAS domains present in WC-1 and WC-2 allow the formation of the White-Collar complex (WCC) (Cheng et al., 2002). The formation of this complex is necessary for the stability of WC-1 (Cheng et al., 2002). The WCC acts as a transcriptional regulator of genes that respond to blue light (Linden et al., 1997). After a brief pulse of blue light, the chromophore covalently binds to a conserved cysteine in the LOV domain of WC-1, causing a conformational change in the protein, promoting binding of the complex to promoters of light responsive genes (Chen et al., 2009).

In *N. crassa*, all responses to blue light are regulated by the White-Collar photoreceptor complex (Linden et al., 1997; Liu et al., 2003). Homologs of the White-Collar proteins have been characterized in *T. atroviride* (Casas-Flores et al., 2004), *Trichoderma reesei* (Castellanos et al., 2010), *Aspergillus nidulans* (Purschwitz et al., 2008), *Phycomyces blakesleeanus* (Idnurm et al., 2006) and in several other fungi distributed in the three great phylogenetic clades of fungi (Ascomycetes, Zygomycetes, and Basidiomycetes) (Corrochano, 2019).

In *N. crassa*, a second blue light photoreceptor, VIVID, a protein with a PAS/LOV domain allows perception of changes in light intensity, triggering adaptive responses (Schwerdtfeger and Linden, 2003). VIVID has been shown to negatively regulate the transcriptional activity of the White-Collar complex (Heintzen et al., 2001; Schwerdtfeger and Linden, 2003; Chen et al., 2010; Malzahn et al., 2010), and plays important roles in the control of the circadian clock in Neurospora (Hunt et al., 2010). Orthologues of *vivid* are found in the genomes of many fungi, including *Fusarium oxysporum*, *Botrytis cinerea*, *T. atroviride* and *T. reesei* (Corrochano, 2019).

The orthologue of *vivid* in *Trichoderma* (*env*-1) codes for the protein ENVOY that plays an important role in light perception (Schmoll et al., 2005). An analysis of the *T. reesei* Δ*env*-1 mutant showed that ENVOY allows the fungus to tolerate continuous exposure to blue light and to perceive changes in light intensity (Castellanos et al., 2010). However, according to Schmoll et al. (2005) it does not complement a *N. crassa vivid* mutant, suggesting different mechanisms of action of these photoreceptors.

In addition to BLR-1 and ENVOY, the *T. atroviride* genome encodes several other potential photoreceptors, such as a phytochrome, an opsin, a 6-4 photolyase (*cry*-1) and a DASH-type cryptochrome (Schmoll et al., 2010; García-Esquivel et al., 2016).

Once light has been perceived, remodeling of the cell’s transcriptional activity begins, either inducing or repressing transcription of genes that control different responses in the organism. In this regard, different microarray and RNA-seq studies have been carried out in fungi with the aim of identifying genes regulated by light. An RNA-seq analysis of gene expression in *N. crassa* allowed the identification of 532 light-responsive genes, with 310 up-regulated and 222 down-regulated (Wu et al., 2014). Similarly, a microarray analysis of genome-wide expression in response to light in *Aspergillus nidulans* led to the identification of more than 400 up-regulated genes and more than 100 down-regulated genes in developmentally competent mycelium (Ruger-Herreros et al., 2011). A recent RNA-seq analysis in *Cordyceps militaris* led to the identification of genes related to carotenoid biosynthesis under light exposure conditions, with 866 up-regulated genes and 856 down-regulated genes (Lou et al., 2019). In *T. reesei*, a microarray analysis allowed the identification of genes responsive to light, of which 137 were up-regulated and 111 were down-regulated in the wild-type strain. Interestingly, in the *T. reesei blr*-1, *blr*-2 and *env*-1 mutants, the number of differentially expressed genes in response to light increased (Tisch and Schmoll, 2013).

In the first studies aiming at the identification of blue-light responsive genes in *T. atroviride* we found both blue light up-regulated (blu) and blue light down-regulated (bld) genes. A microarray analysis containing 1438 unigenes indicated that 2.8% of the genes in *T. atroviride* were responsive to light (Rosales-Saavedra et al., 2006). A more recent study in *T. atroviride*, allowed the identification of 331 genes regulated by white light and 204 by blue light (García-Esquivel et al., 2016).

The viability and fitness of an organism is highly dependent on the integrity of its DNA, as it is prone to damage by many factors. DNA damage can have serious consequences for the cell, which if not repaired leads to the generation of mutations that can be inherited from generation to generation and even cause cell death. Therefore, all organisms have developed sophisticated DNA damage repair mechanisms throughout evolution (Berens and Molines, 2020). In this regard, UV-A radiation (320-400 nm) and UV-B (280-320 nm) are slightly harmful, while UV-C radiation (<280 nm) can be very harmful. However, it is mostly absorbed by ozone and oxygen before it hits the Earth (Sinha and Häder, 2002; Godar, 2005). Exposure to UV-C radiation can cause damage to intracellular macromolecules, which are essential for cell viability and functioning, such as DNA, RNA, proteins, ribosomes and biomembranes (Engelberg et al., 1994; Griffiths et al., 1998). In eukaryotes, UV-A radiation and visible light can indirectly lead to the generation of reactive oxygen species (ROS) such as singlet oxygen and hydroxyl radicals (Aguirre et al., 2005), which, in turn, can damage DNA through indirect photosensitive reactions (Alscher et al., 1997). Furthermore, prolonged exposure to UV radiation generates two main types of DNA damage, cyclobutane pyrimidine dimers (CPD) and (6-4)-pyrimidine-pyrimidine photoproducts (6-4PPs) (Sancar, 2003). These products are toxic to cells as they prevent DNA replication and transcription, resulting in cell death (Süß et al., 2009). Fortunately, cells have several DNA repair pathways, the two main repair pathways are photoreactivation and base excision repair (NER) that allow decomposition of CPDs and 6-4PPs (Suter et al., 2000; Steurer et al., 2019). Photoreactivation is a mechanism that depends on visible light or radiation close to UV, in which photolyases participate (Chaves et al., 2011). In contrast to photoreactivation, NER is a slow and complex mechanism independent of light that requires the participation of many proteins and post-translational modifications (Suter et al., 2000; Marteijn et al., 2014).

Members of the cryptochrome/photolyase family, classified in CPD photolyases, 6-4 PP photolyases, and DASH-type cryptochromes exist in almost all organisms, and filamentous fungi are no exception. In *T. atroviride*, the CPD photolyase, PHR-1, is required for the photorepair of DNA lesions in conidia exposed to UV radiation (Berrocal-Tito et al., 2007). Similarly, in *Beauveria bassiana* and *B. cinerea*, the contribution of CPD photolyases to the repair of DNA lesions induced by UV radiation has been demonstrated (Cohrs and Schumacher, 2017; Wang et al., 2019). In *T. atroviride* and *B. bassiana* the 6-4 PP photolyase CRY-1 and PHR-2, respectively, play an important role in the photorepair of DNA damage induced by UVC light. The DASH-type cryptochrome of *T. atroviride* does not seem to contribute to this process (García-Esquivel et al., 2016; Wang et al., 2019). Interestingly, in *B. cinerea*, the DASH-type cryptochrome (BcCRY2) plays a role as a repressor of conidiation in response to UV and white light (Cohrs and Schumacher, 2017).

In *N. crassa* VIVID plays a negative role in the regulation of the transcriptional activity of the White-Collar complex (WCC). However, this phenomenon has not been explored in *T. atroviride*. Therefore, we decided to study the role of all known blue-light photoreceptors in this process through RNA-seq analyses and phenotypic characterization in *T. atroviride*. We discovered that ENVOY plays a role as a repressor of genes that are induced by light in a BLR dependent manner. Importantly, we also demonstrated that ENVOY is a modulator of the expression of genes related to DNA repair, a discovery of great importance in mycology that could lead to the generation of strains resistant to UV radiation with multiple applications.

## Materials and Methods

### Strains, Culture Medium and Growth Conditions

In the development of this work, the wild type strain (WT) of *T. atroviride* IMI 206040 and the mutant strains Δ*blr*-1 (Casas-Flores et al., 2004), Δ*cry*-*DASH* and Δ*cry*-1 (García-Esquivel et al., 2016), and Δ*env*-1 were used. Pre-inocula were obtained by placing 20 μl of a suspension of conidia (1x10^6^) of the strains on potato dextrose agar (PDA; DIFCO, Detroit, MI), allowing them to grow at 28 °C for 48 hours in the dark. Subsequently, mycelium disks (5 mm in diameter) were placed on Petri dishes containing PDA for photoconidiation and growth tests.

### Phylogenetic Analysis of the ENVOY Protein

The protein sequence of *T. atroviride* ENVOY was structurally compared to lookup sequences via BLASTp analysis online^1^ and aligned with the counterparts found in some representative fungi, followed by a phylogenetic analysis with the neighbor-joining method in MEGA X (Kumar et al., 2018).

### Generation of the Δ*env*-1 Mutant

We replaced the open reading frame (ORF) of the orthologue of *env*-1 (gene Id. 150699) in *T. atroviride* (Supplementary Figure 1) using the Double Joint PCR method, as previously described (Yu et al., 2004; Castellanos et al., 2010). Primers were designed to replace the ORFs of *env*-1 by the selection marker encoding the *Escherichia coli* hygromycin phosphotransferase gene (*hph*) (Supplementary Table 1). This construction was then used for PEG-mediated protoplast transformation of the WT strain as previously described (Castellanos et al., 2010). Nine independent transformants were subjected to three rounds of single spore isolation, and DNA obtained from the purified transformants using standard protocols. Strains that had undergone the gene replacement event were identified by PCR and the one used here for further analysis checked by Southern blot. The response to light of four of these mutants was characterized, all of them showing the same phenotype (Supplementary Figure 2).

### Photoconidiation Tests

For the photoconidiation assays, the strains were inoculated in the center of 100 × 15 mm Petri dishes with 30 ml of PDA medium and grown in the dark at 28°C for 36 h. After this period, the colonies of all the strains were exposed to different blue light fluencies in a PERCIVAL growth chamber model E30LED, illuminated by blue light-emitting diodes (LEDs) (0, 200, 400, 600, 800, 1200 μmol⋅m^–2^), where 0 represents the control kept in complete darkness. Subsequently, all Petri dishes were incubated for an additional 36 hours in the dark. At the end of this period, the Petri dishes were photographed, and conidia collected with 5 ml sterile distilled water. Finally, conidia were quantified in an Axiostar plus, ZEISS optical microscope using a Neubauer chamber (Hemocytometer).

### Photoconidiation Tests at Low and High Intensities of Constant Blue Light

The WT and Δ*env*-1 strains were placed on Petri dishes (100 × 15 mm) containing 30 ml of PDA and allowed to grow at 28°C in a PERCIVAL growth chamber model E30LED, illuminated by blue light-emitting diodes (LED) with a fluence of 2.9 μmol⋅m^–2^⋅s^–1^ (low) or 11.6 μ⋅m^–2^⋅s^–1^ (high). The strains were photographed and the number of conidia per plate determined after 72 h.

### Growth Tests Under Constant White Light and Darkness

Petri dishes (150 × 15 mm) containing 80 ml of PDA were inoculated with the indicated strains in the center and exposed to white light (21 μmol. m^–2^s^–1^) in a programmable air incubator (Model BOD50A16, Revco, Thermo Electron Corp., Asheville) or kept in the dark for 72 h at 28°C. Colony diameter was marked every 12 h to determine the radial growth rate and the total radial growth.

### Growth in Different Carbon Sources

To evaluate the impact of continuous light exposure on carbon-limited growth, the WT and Δ*env-*1 strains were grown on Petri dishes (150 × 15 mm) containing 80 ml Vogel’s minimal medium (VMM) supplemented with 2% agar (Vogel, 1956) and glycerol, sorbose, glucose, fructose or lactose at 2% as sole carbon source, as indicated. The strains were grown in the different carbon sources at a constant blue light fluence of 11.6 μmol.m^–2^s^–1^ for a period of 72 h, with the corresponding controls in the dark.

### Growth Under Photoperiods

For the photoperiod tests, 150 × 15 mm Petri dishes with 80 mL of PDA medium with an inoculum of the WT and Δ*env-*1 strains were grown in a PERCIVAL growth chamber model E30LED, illuminated by blue light-emitting diodes (LED) with a 2.4 μmol⋅m^–2^⋅s^–1^ fluence in successive periods of 12 h light and 12 h darkness during 72 h at 28°C, starting with the light cycle. Radial growth was determined in each cycle.

### Photoreactivation Assays

Five μL drops of a suspension of 4 x 10^4^ conidia⋅ml^–1^ of the WT, Δ*cry*-1 and Δ*env*-1 strains were inoculated on Petri dishes containing PDA supplemented with 0.5% Triton X-100. Conidia were irradiated with UV-C light (254 nm) at a dose of 350 J⋅m^–2^ or 400 J⋅m^–2^ using a Stratalinker^®^ UV crosslinker 2,400. Subsequently, the irradiated conidia were incubated in complete darkness or in constant white light at 28°C for 48 h and colony forming units (CFU) counted and photographed. Controls were treated in the same way but were not UV irradiated.

### Northern Blot Analysis

To evaluate the effect of blue light on gene expression we grew the WT and the Δ*env*-1 strains in the dark for 36 h on PDA plates covered with cellophane sheets and the colonies were incubated in constant blue light (5 μmol⋅m^–2^⋅s^–1^) for 5, 15, 30, 60, 120, 240, or 480 min and collected mycelia. As control, we collected mycelia of colonies that were never exposed to light. After the treatment, mycelial samples were frozen in liquid nitrogen and used for RNA extraction.

Total RNA was extracted using the TRIzol protocol (Invitrogen). The Southern and Northern hybridizations were carried out using conventional methods (Sambrook and Russell, 2001).

Radioactive labeling of the probes with [α-32P]-dCTP was carried out by the “Random Priming” method, following the supplier’s protocol (Amersham Biosciences). Probes were obtained by PCR amplification from DNA of the WT strain, using the INVITROGEN^®^ Recombinant Taq method and reagents. Subsequently the probes were purified using columns by the QIAGEN^®^ method, cutting the corresponding band of the agarose gel, obtained by electrophoresis.

### RNA-seq and Differential Expression Analysis

Mycelium of the WT, Δ*blr*-1, Δ*env*-1, Δ*cry-DASH* and Δ*cry*-1 strains were collected 30 min following a blue light pulse of 200 μmol⋅m^–2^ and immediately frozen in liquid nitrogen. A set of plates of all the strains was not exposed to light and used as control. RNA libraries were prepared with the TruSeq v2 RNA Sample Preparation Kit (Illumina). The size and quality of the library were determined using a Bioanalyzer (Agilent Technologies). Three biological replicates of all conditions were sequenced on an Illumina HiSeq 2,500 platform with an average of 20 million reads per library. Read quality was analyzed using the FastQC software^2^. High-quality reads were then mapped to the predicted transcripts of the *T. atroviride* v2 genome^3^ using the Kallisto software (Bray et al., 2016). The result of the table count obtained with Kallisto was used to construct an expression matrix of the light-dark conditions of all strains of *T. atroviride*. The expression matrix generated was used for differential expression analysis using R-Studio and the *edgeR* (Robinson et al., 2009) and *limma* (Ritchie et al., 2015) packages. The count matrix was normalized using the M-values (TMM) method (Robinson and Oshlack, 2010). Differentially expressed genes were determined using the Generalized Linear Probability Radius Test (GLM) method with a Log Fold-Change of ± 1 and a FDR ≤0.05. The RNA-seq data analyzed in this publication have been deposited with the NCBI’s Gene Expression Omnibus and can be accessed through the GEO series accession number GSE165935^4^.

### Annotation and Enrichment Analysis of GO Terms

Differentially expressed genes were annotated using a data matrix generated by Blast2GO (Conesa et al., 2005) and the enrichment analysis of functional categories was performed in R-Studio of biological processes using Camera from the *limma* package (Wu and Smyth, 2012). GO terms with FDR ≤0.05 were considered significantly enriched in each comparison.

## Results

### ENVOY Is a Master Modulator of the Light Control of the Growth/Conidiation Balance

**FIGURE 1:**
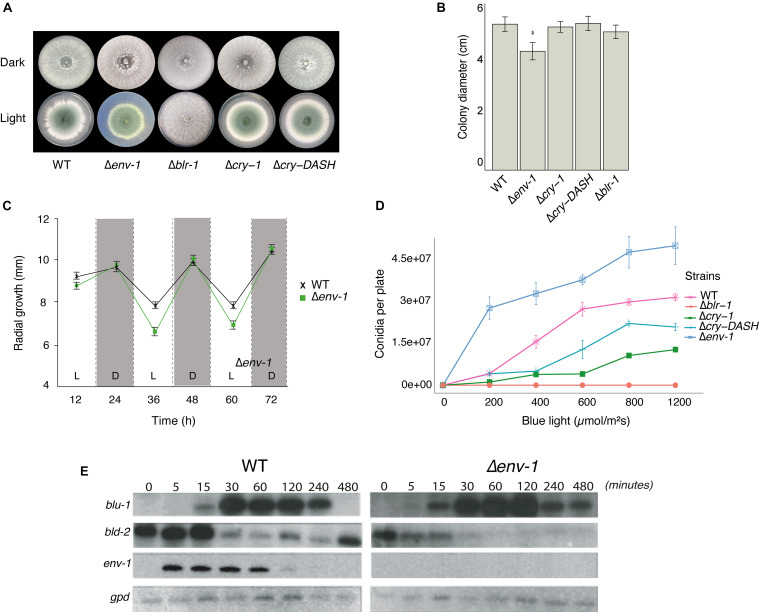
Effect of light on growth and conidiation in the WT strain and the mutants Δ*blr*-1, Δ*cry*-1, Δ*cry-DASH* and Δ*env*-1. **(A)** Phenotype of the strains growing under darkness and constant light exposure. **(B)** Total growth of colonies under constant light exposure. Growth was measured every 12 h for 72 h under constant light and darkness on potato dextrose agar (PDA). **(C)** Radial growth of the WT and Δ*env*-1 strains under 12-h light/dark cycles with 2.9 μmol⋅m^– 2^⋅s^– 1^ of blue light. **(D)** Production of conidia at different fluencies of blue light. The strains were grown for 36 h in the dark, then exposed to a pulse of blue light of 0, 200, 400, 600, 800, and 1,200 μmol⋅m^–2^. Bars show means ± SEM. **(E)** Expression of the *blu*-1, *bld*-2 and *env*-1 genes under constant illumination (5 μmol.m^–2^s^–1^) at times 5, 15, 30, 60, 120, 240, and 480 min. Asterisks indicate statistically significant differences (*P* < 0.05; *n* = 8). A simple ANOVA analysis was used for each condition.

Light has a negative impact on mycelial growth in *T. atroviride*, *Tuber borchii* and *N. crassa*, which requires the White-Collar proteins (Ambra et al., 2004; Casas-Flores et al., 2004; Esquivel-Naranjo and Herrera-Estrella, 2007). We evaluated the effect of continuous exposure to white light on colony growth of the WT strain of *T. atroviride*, and the Δ*blr*-1, Δ*env*-1, Δ*cry-DASH* and Δ*cry*-1 mutants in PDA. We did not find statistically significant differences in the growth of these strains in the dark (*P* < 0.05; Figure 1A and Supplementary Figure 3). However, under constant illumination the Δ*env*-1 mutant showed a 20% reduction in growth compared to the WT strain (Figure 1B). Based on this observation, we decided to evaluate the effect of continuous exposure to blue light on the growth of the WT strain and the Δ*env*-1 mutant using low (2.9 μmol⋅m^–2^⋅s^–1^) and high (11.6 μmol⋅m^–2^⋅s^–1^) light intensities. When the strains were exposed to low light intensity, we observed a more pronounced negative effect in the Δ*env*-1 mutant than in the WT (Supplementary Figure 4). At high intensity, we observed a decrease in growth in the Δ*env*-1 mutant like that observed at low intensity. (Supplementary Figure 5). These results indicate that ENVOY plays an important role in regulating growth under constant illumination in *T. atroviride*. We also found that the ENVOY plays a major role in adaptation to light, since in experiments in which the mutants were grown in 12 h light/12 h dark cycles in PDA it was evident that during exposure to light their growth strongly slowed down and accelerated during the dark cycle, which was much more evident in the mutant strain (Figure 1C).

Despite the drastic decrease in mycelial growth caused by exposure to light, there was a marked increase in conidia production in the Δ*env*-1 mutant (Figure 1A and Supplementary Figures 4, 5). This led us to think that ENVOY could play a role as a central regulator of the growth/development balance, establishing thresholds and controlling excessive responses to intense or constant light stimuli. To delve into this, we evaluated conidia production using different doses of light for the different strains under study in PDA medium. The production of conidia by the WT strain was directly proportional to the dose of light, reaching saturation at 600 μmol⋅m^–2^ (Figure 1D). This is likely due to the existence of a genetic program that modulates the intensity of the response to manage nutrients and energy upon reaching a certain threshold. Similarly, the Δ*cry*-1 and Δ*cry-DASH* mutants, produced conidia in direct proportion to the light dose received, although conidia production was reduced compared to the WT, and required 800 μmol⋅m^–2^ to reach saturation. As expected, the Δ*blr*-1 mutant did not respond to the light stimulus, regardless of the dose applied (Casas-Flores et al., 2004). In contrast, the Δ*env*-1 mutant showed greater production of conidia compared to the other strains, a trend observed at all light dosages applied and did not reach saturation even upon exposure to fluencies greater than 1200 μmol⋅m^–2^. This indicates that ENVOY is a photoreceptor that functions as the central modulator of the growth/conidiation balance in the response to light.

### ENVOY Is a Modulator of Gene Expression in Response to Blue Light and Essential for Photoadaptation

Photoadaptation is the ability of an organism to dim the response to light and prepare it to respond to a second pulse of light. To evaluate the role of ENVOY in the response to light of *T. atroviride*, and in photoadaptation, we selected as reporters the genes *blu*-1 (Id. 300570, orthologue of the *N. crassa grg*-1) and *bld*-2 (Id. 301399, encoding a short-chain dehydrogenase reductase), due to their strong response to light. We evaluated their expression upon continuous exposure to blue light, as well as the expression of *env*-1 in the WT strain and the Δ*env*-1 mutant in a time course experiment. Overall, we observed that *blu*-1 reaches higher levels in the Δ*env*-1 strain than in the WT (Figure 1E). In the WT strain expression of *blu*-1 is detectable 15 min after exposure to light, reaches its maximum by 30 min and then starts decreasing until it disappears (240 min). In contrast, in the Δ*env*-1 mutant, the *blu*-1 transcript was detected already after 5 min, increasing its levels until reaching a maximum by 120 min and could still be detected after 480 min. These observations suggest that ENVOY attenuates the expression of *blu*-1. For the *bld*-2 gene, the expression levels in the dark and in the first few minutes after exposure to light were clearly higher in the WT than in the Δ*env*-1 mutant (5-15 min). By 30 min the expression of this gene decreased to its minimum in the WT strain, while a strong decrease was observed already 5 min after exposure of the mutant to light. In the WT strain the level of expression of *bld-*2 remained at about the same level for at least 210 min and started increasing by 480 min. However, the Δ*env*-1 mutant reached its minimal level (almost undetectable) within 30 min of exposure to light and did not increase after 480 min (Figure 1E). These results provide further support for the proposed role of ENVOY as a negative modulator of the impact of light on gene regulation and show that the expression of both *blu*-1 and *bld*-2 is subjected to a photoadaptation process which is lost in the Δ*env*-1 mutant strain.

### Genes Involved in Conidia Formation Are Regulated Early by ENVOY

**FIGURE 2:**
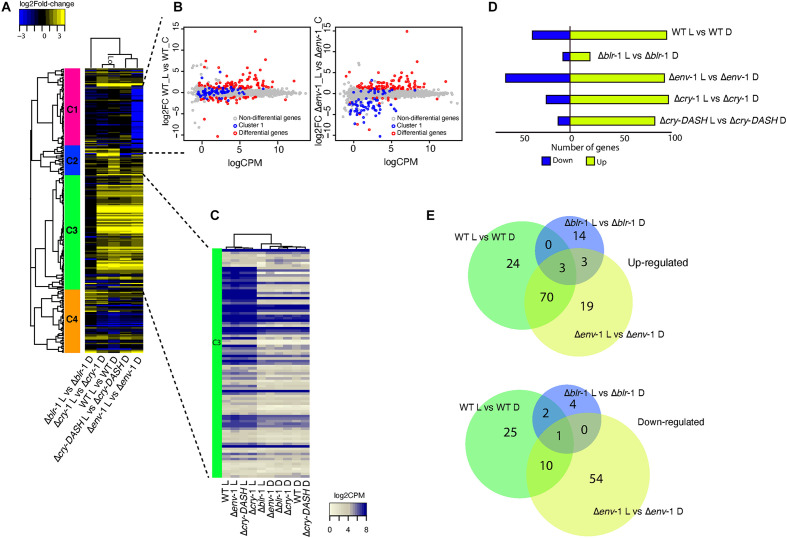
Transcriptional profile of the WT strain and the mutants Δ*blr-*1, Δ*cry-*1, Δ*cry-DASH* and Δ*env-*1 in response to a 200 μmol⋅m^–2^⋅s^–1^ pulse of blue light. **(A)** Heatmap of genes responsive to blue light. Colored bars with roman numerals on the left side of the heatmap indicate gene clusters differentially expressed in the WT and photoreceptor mutants. The scale bar at the top left of the heatmap indicates the gene expression intensity and the lines that support the groups formed in the heatmap are the dendrogram. **(B)** Smear plot generated from edgeR showing the log2 fold change (FC) against the average log count per million (CPM) of genes that respond to blue light in WT strain and Δ*env*-1 mutant. **(C)** Heatmap of log count per million (CPM) values for genes exposed to blue light and kept in dark from cluster three. **(D)** Comparison of the expression profile of the genes differentially expressed between each strain. A Fold-Change filter of ± 1 and a FDR ≤ 0.05 was used. Strains growing in darkness were used as a control. **(E)** Venn diagrams showing the number of up- and down-regulated genes shared by the WT strain and the Δ*blr-*1 and Δ*env*-1 mutants or that are unique for each contrast.

Since blue light triggers transcriptional changes in *T. atroviride* (Rosales-Saavedra et al., 2006) and due to our previous observation on the Δ*env*-1 mutant, we decided to perform high-throughput RNA sequencing analysis of the WT, Δ*blr*-1, Δ*cry*-1, Δ*cry-DASH* and Δ*env*-1 strains with the aim of identifying genes responsive to blue light and the receptor involved (Figure 2). As previously described, we used a low dose of blue light (200 μmol⋅m^–2^) because this is the threshold for photo-conidiation in the WT and we assumed the observed effect would only be due to photoperception and not an indirect effect through reactive oxygen species. We collected mycelial samples of the different strains 30 minutes after the light pulse for the RNAseq experiment. A differential expression analysis of the data allowed us to identify 135 genes responsive to light in the WT (Supplementary Table 2), 27 in Δ*blr*-1 mutant (Supplementary Table 3), 123 in Δ*cry*-1 (Supplementary Table 4), 97 in Δ*cry-DASH* (Supplementary Table 5) and 160 in Δ*env-*1 (Supplementary Table 6 and Figure 2D). Within the set of differentially expressed genes in the WT strain, we found *blu*-1 (*grg*-1), *blu*-2 (*phr*-1), *blu*-4, *al*-3, *cry*-1, *env*-1, *bld*-2 and *bld*-4, previously reported as blue light responsive genes, among others. In agreement with previous results, the transcripts of *blu*-1, *blu*-4, *phr*-1, *al*-3, *cry*-1 and *env*-1 increased their level, while those of *bld*-2 and *bld*-4 were strongly reduced (Rosales-Saavedra et al., 2006; Esquivel-Naranjo and Herrera-Estrella, 2007; García-Esquivel et al., 2016).

An interesting observation was the drastic change in the number of differentially expressed genes in the Δ*env*-1 mutant, particularly an increase in the number of genes repressed in response to light (Figure 2D). Cluster analysis of the heat-map shown in Figure 2A indicated a group of genes (cluster 1) repressed only in the Δ*env*-1 mutant. When we examined the behavior of these genes in a scatterplot, in the contrasts WT-Light vs. WT-Control and Δ*env*-1-Light vs Δ*env*-1-Control, we observed that these genes remained without apparent change in the WT strain in response to the stimulus. However, in the Δ*env*-1 mutant their expression level decreased. When looking at the reads associated with these genes, we found that in the WT strain their expression was low and highly variable. Consequently, they were not classified as differentially expressed, while in the Δ*env*-1 mutant they had a constant basal expression level in darkness, which decreased slightly in response to blue light. Interestingly within the genes enclosed in this cluster we found one that encodes a hydrophobin (Id. 258295) with a log2 Fold-change (FC) of -4.1396, the conidial pigment polyketide synthase *alb*-1 (Id. 217154) with a log2 FC of −3.84 (Gerin et al., 2018; He et al., 2018) and the conidiospore surface protein *cmp*-1 (Id. 323283) with a log2 FC of −3.67 (Puyesky et al., 1999). These genes are closely linked with the late stages of conidia formation and as expected, they did not seem to undergo changes in their expression level and maintained low levels early after exposure to light in the WT strain. Interestingly, these genes were already activated in the Δ*env*-1 mutant and they began to vary in their expression very early in response to light. This observation suggests that cells are hypersensitive to light in the absence of *env*-1 and that this cue acts as a priming signal for the expression of genes that will participate in conidiation several hours later, when other developmental signals concur.

### BLR-1 Regulates Most of the *T. atroviride* Blue-Light Responsive Genes

An evident observation was the reduced number of differentially expressed genes in response to light in the Δ*blr*-1 mutant (Figure 2D). When analyzing the behavior of the genes of cluster III in the heat-map (Figure 2A), it was notable that in the WT strain and the mutants Δ*cry*-1, Δ*cry-DASH* and Δ*env*-1, a large part of these genes were up-regulated in response to blue light. In contrast, in the Δ*blr*-1 mutant they were not, behaving like in the rest of the strains kept in darkness (Figure 2C), thus confirming that BLR-1 is the main regulator of the transcriptional response to blue light. The expression level of these genes does not seem to depend on any other photoreceptor, so all functions played by them, including those of ENVOY, are modulated at the transcriptional level by BLR-1.

The Venn diagram analysis showed that 96% of the genes (94 genes) that are induced by blue light in the WT strain depend on BLR-1. Interestingly, within these genes we found that *env*-1 was strongly induced (Id. 150699; FC: 57.95), despite the low light-dose used in our experiments. The expression of other genes encoding photoreceptor proteins were also in this group, including *cry*-1 (Id. 86846; FC: 3.58), *cry*-DASH (Id. 285589; FC: 4.23), and *phr*-1 (Id. 302457; FC: 2.5). We also observed that the orthologue of frequency (Id. 131340), encoding the master circadian rhythm regulator is induced via BLR-1 (FC: 4.76) at the same time and dose as *tmk*-3 (Id. 301235; FC: 2.67). Thus, all known potential blue-light photoreceptor encoding genes and key elements in the regulation of the circadian rhythms are under the regulation of BLR-1 (Supplementary Table 7). Using the same approach, we found that 92% of genes (35 genes) that are repressed by blue light in the WT strain depend on Blr-1, within which there is a gene that codes for a carboxylic acid transport protein (Id. 29031), another for a terpenoid synthase (Id. 298910), and one for a polyketide synthase (Id. 45973), among other genes (Figure 2E and Supplementary Table 7). These results show that BLR-1 is the central photoreceptor within the light perception system in *T. atroviride*.

In the absence of the BLR-1, there are some genes that respond to blue light and that also respond in the Δ*env*-1 mutant. Among this set of genes we found induced genes encoding hypothetical proteins (Id. 31436, 291965, and 301968) and a gene encoding an alcohol oxidase that is repressed (Id. 81139). These genes could be regulated by another photoreceptor different from the ones analyzed here. Additionally, there are only two genes negatively regulated by blue light in the WT strain that were still responsive in the Δ*blr*-1 mutant, which encode a hypothetical protein and a STF2-like protein (Id. 301901). This confirms that the main photoreceptor is BLR-1 since this number of genes is insignificant, compared to the genes that are no longer responsive in the *blr*-1 mutant.

### ENVOY Modulates the Expression of BLR-1 Dependent Genes

Given the modulatory role of ENVOY on growth and conidiation, we analyzed its influence on the expression of genes responsive to light in a BLR-1 dependent manner in the WT strain. We determined that 70 positively regulated genes are shared between the WT and the Δ*env*-1 mutant, of which 64% (45 genes) present a higher level of expression in the Δ*env*-1 mutant than in the WT (Figure 2E and Supplementary Table 8). It is noteworthy that within this group of genes we found a gene that codes for a DNA excision repair protein (Id. 26345), another for the CPD-photolyase PHR-1 (Id. 302457), one for an RNA exonuclease (Id. 218054), another for the MAPK TMK3 (Id. 301235), a protein linking light and stress responses (Esquivel-Naranjo et al., 2016) and to the orthologue of the *N. crassa frq* gene (Id. 131340), which participates in the regulation of circadian rhythms (Cha et al., 2014). Interestingly, in the Δ*env*-1 mutant we found reads aligning to the 3’ untranslated region of *env*-1, which was not affected by the gene replacement event, indicating that ENVOY is not necessary for its own induction (Supplementary Figures 2, 6 and Supplementary Table 6). These results are consistent with the role of ENVOY in photoadaptation of the light responses regulated by BLR-1. For the genes that are repressed and shared between the WT and Δ*env*-1, nine out of ten genes are less repressed in the Δ*env*-1 mutant than in the WT (Figure 2E and Supplementary Table 8). Within these nine genes, we identified a gene encoding a translocation protein Sec62 (Id. 33428), another a FAD monooxygenase (Id. 36860), one for an MFS monocarboxylate transporter (Id. 153853), another for a short-chain dehydrogenase reductase *sdr* (Id. 301399) and a gene coding for a fungal transcriptional regulatory protein (Id. 87055), among others.

### ENVOY and CRY*-*1 Are Involved in the Regulation of Carbon and Nitrogen Metabolism in Response to Light

**FIGURE 3:**
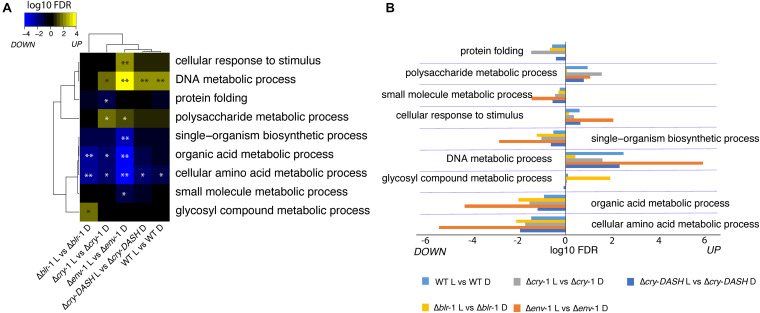
Enrichment analysis biological processes in response to blue light in the WT strain and the photoreceptors mutants. **(A,B)** Significantly enriched Gene Ontology (GO) terms related to DNA, carbon and nitrogen metabolism in response to blue light in the indicated strain (^∗^FDR < 0.05; ^∗^FDR < 0.01). Each block **(A)** and horizontal bar **(B)** contains the up and down regulated functional categories.

To determine which biological processes are affected in *T. atroviride* in the response to blue light, we performed an enrichment analysis of functional categories based on our transcriptomic data. This analysis showed that blue light affects many processes related to primary carbon and nitrogen metabolism (Figures 3A,B). In this regard, the expression of genes involved in single-organism biosynthetic processes and small molecule metabolic processes were affected by light, in all strains analyzed. However, in the Δ*env*-1 mutant they were significantly depleted. Similarly, we observed that the expression of genes that participate in biosynthetic processes of organic acids and amino acids was affected by exposure to blue light; this effect was more pronounced in the Δ*env*-1 mutant. Interestingly, we observed that the same genes that are enriched in Δ*env*-1 are also enriched in the Δ*cry*-1 mutant. It was particularly interesting to see that in the absence of either ENVOY or CRY-1, there is an enrichment in the response to light of polysaccharide metabolism processes and a depletion of organic acid and amino acid metabolic processes (Figures 3A,B). This suggests that these two genes, in addition to being important for DNA metabolism, play a role in controlling the metabolic state of the cell. Furthermore, it indicates that to respond to stress by activating DNA metabolism and response to stimuli, a modification of the central metabolism of cells is necessary and this change appears to be mediated by the ENVOY and CRY-1 photoreceptors.

### ENVOY Is Determinant for the Efficient Use of Carbon Sources During the Response to Light

**FIGURE 4:**
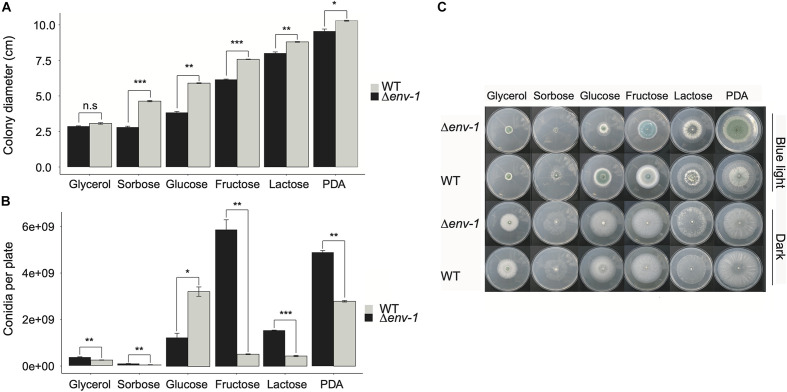
Development and growth of the WT and Δ*env*-1 mutant on different carbon sources. **(A)** Variation in the growth of the WT strain and the Δ*env*-1 mutant in media with different carbon sources. **(B)** Variation in the conidiation of the WT strain and the Δ*env*-1 mutant in media with different carbon sources. **(C)** Growth phenotype of the WT strain and the Δ*env*-1 mutant grown on different carbon sources and under constant light and darkness. A *t*-test was used to determine the significant differences between each pair of strains for each carbon source, indicated by asterisks according to the significance (*P* > 0.05; no significant difference; n.s., *P < 0.05, **P < 0.01, ***P < 0.001; *n* = 9).

Considering the observations made in the analysis of functional categories, we decided to evaluate the effect that different carbon sources, in combination with continuous illumination, have on the growth of the WT strain and the Δ*env*-1 mutant. We observed that both strains utilize differently the carbon sources tested, which results in poor growth in some carbon sources, compared to their growth in PDA medium (Figures 4A-C). A greater decrease in growth was observed in glycerol, although both strains had a very similar growth deficit. A similar effect was observed when using sorbose as sole carbon source. However, in this case, the Δ*env*-1 mutant grew significantly less than the WT strain (*t*-test; *P* < 0.001). Glucose and fructose had a similar effect on the growth of the fungus, the effect was greater when glucose was the only carbon source, with a stronger impact of light on the growth capacity of the Δ*env*-1 mutant in both carbon sources. Finally, we observed a slight defect in the growth of both strains when lactose was the sole carbon source, compared to PDA medium (Figures 4A,C). These results demonstrate that the Δ*env*-1 mutant strain decreases its efficiency in the utilization of various carbon sources when exposed to light, which is consistent with the depletion in the expression of genes involved in carbon metabolic processes. However, it was interesting to observe a hyperconidiating phenotype in the Δ*env*-1 mutant, producing practically more than twice as many conidia as compared to the WT strain in media supplemented with lactose, fructose, and even sorbose. Although the production of conidia was almost negligible in sorbose. When the Δ*env*-1 mutant grew on fructose as sole carbon source, the increase in conidia production was 11-fold, compared to the WT strain. Curiously, in the medium with glucose, we observed the opposite effect, since the mutant strain produced approximately half the conidia produced by the WT strain (Figure 4B). Based on these results, we propose that ENVOY is a link between nutrient and light signaling.

**FIGURE 5:**
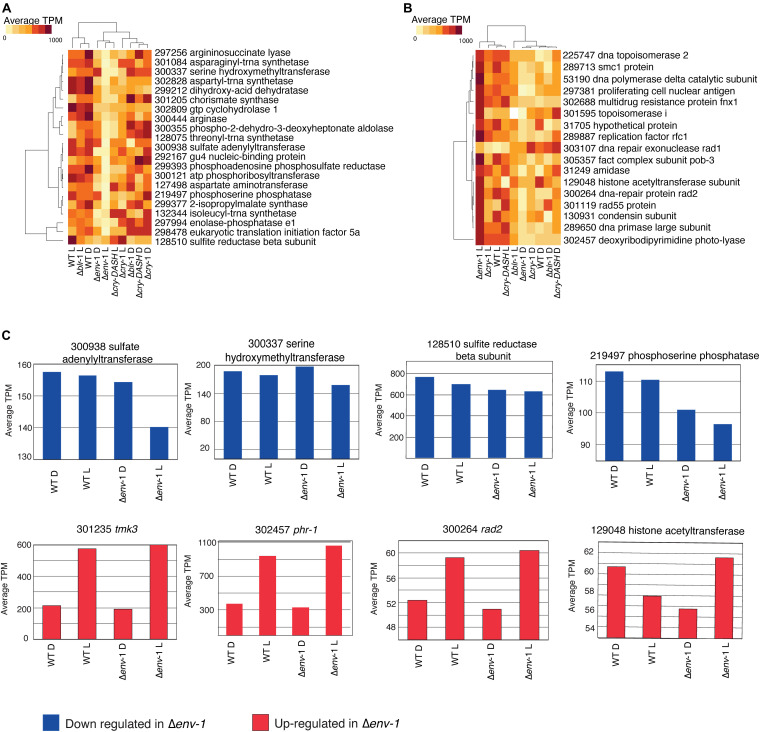
Expression of genes related to amino acid metabolism and DNA repair are affected by the ENVOY mutation. **(A)** Expression profile of the genes contained in the category of cellular amino acid metabolic process, which have a repressive behavior in Δ*env-*1 in response to light. **(B)** Expression profile of the genes contained in the category of DNA metabolic process, which have an induction behavior in Δ*env*-1 in response to light. **(A,B)** Colors indicate the average counts per million. **(C)** Bar graphs of the average counts per million of genes repressed and induced in response to light in the WT strain, which show a drastic change in the Δ*env*-1. Blue bars represent down-regulated genes and red bars up-regulated genes.

### CRY*-*1 and CRY-DASH Contribute to the Photoconidiation Response but Are BLR-1 Dependent

Our transcriptomic data showed that *phr*-1, *cry*-1 and *cry-DASH* are induced by blue light in a BLR dependent manner. Thus, we analyzed the role of CRY-1 and CRY-DASH in the physiological responses of *T. atroviride*. One of the main responses to light of *T. atroviride* is the production of conidia after exposure to a light pulse. In this sense, our results showed a contribution of the Cry-1 and Cry-DASH in this photoresponse, since mutants in either gene produced less conidia than the WT strain (Figure 1D). In addition, we evaluated the effect of blue light on mycelial growth in the Δ*cry*-1 and Δ*cry-DASH* mutants. However, we did not observe significant differences in growth compared to the WT strain, which suggests that these photoreceptors do not intervene in the light dependent control of nutrient utilization and growth control (Figure 1B).

Additionally, we decided to evaluate whether CRY-1 and CRY-DASH play a relevant role in the light dependent regulation of gene expression in *T. atroviride*. Using Venn diagrams, we identified genes regulated by these photoreceptors, finding 26 up-regulated genes that are CRY-1 dependent. Within this set of genes, we found one that encodes a cytochrome p450 (Id. 129185), another for an autophagy protein (Id. 40111), one for a phospholipase D (Id. 34379) and one for C2H2-type Zinc finger protein (Id. 87968). Additionally, we found 20 CRY-1 dependent down-regulated genes, among which is a gene that codes for a carboxylic acid transport protein (Id. 29031), one for a glycoside hydrolase family member (Id. 31864), another for an aromatic amino acid aminotransferase (Id. 45548) and a gene encoding a polyketide synthase (Id. 45973), among other genes (Supplementary Figure 7 and Supplementary Table 9). On the other hand, we found 27 CRY-DASH dependent up-regulated genes, within which there is a gene that codes for a fungal transcriptional regulatory protein (Id. 41299), and one for a DCR-like BAHD acyltransferase (Id. 94131), among others. Also, 29 down-regulated genes were CRY-DASH dependent (Supplementary Figure 7). In this group of genes, we found a gene that encodes a major facilitator superfamily transporter (Id. 45374), another an integral membrane protein (Id. 85568), and a gene coding for a lipase 3 precursor (Id. 317495), among others (Supplementary Table 10). Importantly, some genes down-regulated in both mutants are related to carbon and nitrogen metabolism, which suggests that these photoreceptors play an important role in this process. Interestingly, we found 24 up-regulated and 20 down-regulated genes regulated by both photoreceptors (Supplementary Figure 7).

### Genes Involved in the Metabolism of Sulfur-Containing Amino Acids Are Regulated by ENVOY

The expression of some genes related to amino acid biosynthesis decreased in response to blue light in the Δ*env*-1 mutant with respect to the rest of the strains (Figures 5A,B), such as a gene that encodes for a sulfate adenylyl transferase (Id. 300938), another for a serine hydroxymethyltransferase (Id. 300337), another for a phosphoserine phosphatase (Id. 219497) and yet another for a sulfite reductase beta subunit (Id. 128510), as well as genes that encode enzymes important in the biosynthesis of adenylyl sulfate and sulfur amino acids, which is congruent with the depletion in categories related to nitrogen metabolic processes (Figure 5C). In general, many processes related to metabolism of sulfur-containing amino acids were more depleted in response to light in the Δ*env-*1 mutant than in all other strains tested (Supplementary Table 5). This metabolic arrest may explain in part the defect in the growth of the Δ*env*-1 mutant in response to light.

On the other hand, the genes contained in the DNA metabolism category seem to increase their expression consistently in the Δ*env*-1 mutant (Figure 5B), strongly suggesting that the said mutant may have the genetic material repair system in a more active state.

### ENVOY Negatively Regulates DNA Repair

As described above, in the Δ*env*-1 mutant we observed a drastic enrichment of DNA repair processes (Figure 3). In this regard, Esquivel-Naranjo et al. (2016) observed that both *pbs*-2 and *tmk*-3 play an important role in photo-repair. Along with *tmk*-3, *phr*-1, and *cry*-1, other genes categorized within DNA metabolic processes appeared to be more responsive to light in the *env*-1 mutant. Thus, seventeen genes categorized as involved in DNA metabolic processes were selected and their expression visualized as CPM (Figure 5B). Interestingly, all 17 genes showed a higher expression level in the Δ*env-*1 mutant than in the WT strain and the other mutants. Within this group of genes, some code for DNA repair and DNA replication checkpoint proteins during the cell cycle such as those encoding the Rad-type proteins: RAD-1 (Id. 303107), RAD-2 (Id. 300264) and RAD-55 (Id. 301119) (Murakami and Okayama, 1997), one encodes the photolyase PHR-1, involved in the repair of pyrimidine cyclobutane dimers (CPDs) (Berrocal-Tito et al., 2007), one more a DNA polymerase (Id. 53190), one a histone acetyltransferase type B subunit 2 (Id. 129048), involved in the acetylation of newly synthesized histones during chromatin assembly processes (Parthun, 2007), and two topoisomerases (Id. 225747 and 301595). These results indicate a repressive role of the genetic material repair processes by ENVOY. We did not observe this expression pattern when the Δ*env*-1 mutant was grown in the dark (Figure 5B), which shows that this behavior is blue light dependent.

### The Δ*env*-1 Mutant Repairs DNA More Efficiently

**FIGURE 6:**
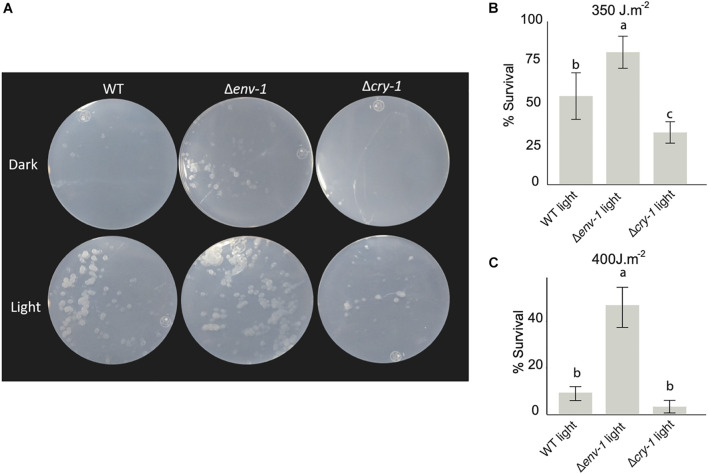
*env*-1 mutation has a positive effect on photoreactivation. **(A)** Colonies of the WT strain and the Δ*cry*-1 and Δ*env*-1 mutants incubated for 48 hours in constant light and dark conditions after being irradiated with 350 J⋅m^–2^ or 400 J⋅m^–2^ UV-C light. **(B,C)** Survival of the colonies of the photoreactivated strains at 350 J⋅m^–2^ **(B)** or 400 J⋅m^–2^ **(C)**. Colonies were counted after incubation in light and the results were represented as percent survival for each condition relative to the control not irradiated with UV-C light. One-way ANOVA (Tukey’s Post Hoc Test with 95% confidence interval) was used for each condition. Bars show means ± SD. The letters above each bar indicate statistically significant differences (*P* < 0.05; *n* = 9).

Because the transcriptomic analysis strongly suggested that the Δ*env*-1 mutant could have a higher DNA repair capacity, given that genes encoding proteins involved in photorepair systems were overstimulated, we decided to carry out photoreactivation assays. In the WT strain survival upon photoreactivation was 55% and only 11% in darkness. In the Δ*cry*-1 mutant, survival upon photoreactivation was reduced to 32%, and had a survival of 9% in darkness (Figures 6A,B). These results agree with previous reports for *T. atroviride* and *T. reesei* mutants in *cry*-1, which possess 6-4 photolyase activity (Guzmán-Moreno et al., 2014; García-Esquivel et al., 2016). Interestingly, in our photoreactivation experiments, we observed 82% survival when the Δ*env*-1 mutant was exposed to visible light after being subjected to UV irradiation (photoreactivation) and only 19% survival when not exposed to visible light. This contrasts with the 55% survival observed for the WT strain upon photoreactivation, which implies that the transcriptional response is reflected in a functional phenotype of higher tolerance to UV-light. When we increased the dose of ultraviolet light to 400 J⋅m^–2^, the survival rate after photoreactivation decreased dramatically in all strains, but the difference between the Δ*env*-1 mutant and the other strains was even more evident than at 350 J⋅m^–2^ (Figure 6C). It is worth mentioning that even when not exposed to white light after the UV treatment the Δ*env*-1 mutant showed higher tolerance to UV irradiation (Figure 6A). This might be the result of the induction of *phr*-1 and *rad*-2 as a result of the exposure to the light source used for the UV treatment that chiefly emits 254nm light but may also emit in the UV-B range, which has been shown to slightly induce conidiation (Kumagai and Oda, 1969). Thus, ENVOY functions as a repressor of DNA repair by controlling the level of expression of the genes involved in this process (Figure 6 and Supplementary Figure 8).

## Discussion

**FIGURE 7:**
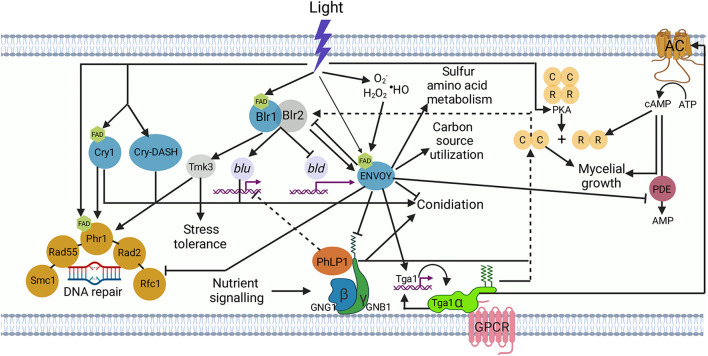
*Trichoderma atroviride* light signal perception and transduction. The BLR-1 and BLR-2 proteins sense blue light through the chromophore attached to the BLR-1 protein, a flavin adenine dinucleotide molecule (FAD; pentagon; Casas-Flores et al., 2004). The activated BLR complex binds to the promoters of target genes, leading to the activation of blue light up-regulated (*blu*) or repression of blue light down-regulated (*bld*) genes. The increase in the proteins encoded by the *blu* genes leads to stress tolerance, DNA repair, conidiation, and the synthesis of proteins of secondary photoreceptors, including CRY1, CRY-DASH, and ENVOY. Once ENVOY perceives light, presumably through FAD (Schmoll et al., 2005), it modulates *blu* and *bld* gene expression by putatively inactivating the BLR complex, resulting in the activation of carbon source utilization and sulfur metabolism genes, promotion of mycelial growth, and repression of DNA repair genes and conidiation. Besides its activation by light ENVOY perceives reactive oxygen species (H_2_O_2_, O_2_•^–^ and •HO) provoked by the interaction of light with biological molecules such as flavins and porphyrins, or by nutrient deprivation (Lokhandwala et al., 2015). Light stimulates protein kinase A (PKA) activity (Casas-Flores et al., 2006). ENVOY represses the activity of the phosphodiesterase (PDE); an enzyme that degrades cAMP accumulated owing to the activation of TGA1, which activates adenylyl cyclase (AC), allowing the synthesis of cAMP (cyclic adenosine monophosphate). Accumulation of cAMP leads to the release of the catalytic subunit (C) of protein kinase A (PKA) due to binding of cAMP to the regulatory subunit (R), to positively regulate conidiation and mycelial growth. PKA could also positively regulate the BLR complex (dashed lines from PKA to BLR complex). The accumulation of cAMP has a positive effect on mycelial growth. ENVOY negatively regulates the putative interaction of the phosducin-like (PhPL chaperone) product with the GNB1/GNG1 heterodimer to dissociate the active complex that positively regulates conidiation (Tisch et al., 2014). Nutrient signaling has a positive effect on the PhPL/GNB1/GNG1 complex (Tisch et al., 2014). The PhPL/GNB1/GNG1 complex exerts a negative regulation on *blu* genes. In the inactive state, the three G-protein subunits (α, β, γ) are present in a complex, in association with G-protein-coupled receptors (GPCRs). Ligand binding to the GPCR leads to dissociation of the Gα subunit from the Gβγ dimer. Solid arrows represent positive regulation, whereas dotted arrows show a presumably positive regulation. Lines with a bar at the end indicate negative regulation.

In the filamentous fungi, *N. crassa* and *T. reesei*, the function of the *vivid/env*-1 gene in their photoresponses has been studied. Although the dependence of the WCC system and the participation as photoreceptors of VIVID/ENVOY in these systems is evident, until now it is unclear why they appear to exert different functional roles, since expression of *env*-1 in a *N. crassa vivid* mutant does not restore the WT phenotype. Here, we characterized at the morphological and transcriptional level mutants in the main blue light photoreceptors identified in the *T. atroviride* genome, with special attention to the orthologue of *vivid/env-*1 (see Supplementary Figure 1). This allowed us to find genes regulated by each of the photoreceptors and to determine that in *T. atroviride* ENVOY effectively depends on the activity of BLR-1 and that like VIVID in *Neurospora*, plays critical a role as a negative regulator of the response to light. Furthermore, we found that ENVOY regulates growth, central metabolism, conidiation, and DNA repair in *T. atroviride* (see model in Figure 7). An interesting observation during the development of this work was that deletion of *env*-1 results in the exacerbated production of conidia when grown under continuous illumination. Even at high light doses, the Δ*env*-1 mutant does not reach a plateau in conidia production, which is reached in the WT strain at 600 μmol⋅m^–2^. This observation indicates that *env*-1 is essential to determine the amount of light required to saturate the response to light. On the other hand, the Δ*env*-1 mutant shows a drastic growth defect when facing constant light, like that reported in *T. reesei* for a mutant in the orthologous gene (Schmoll et al., 2005; Castellanos et al., 2010). Given all the above, our first conclusion is that ENVOY modulates the balance between growth and conidiation in *T. atroviride* (Figure 7).

In this context, a first experiment to characterize the expression profile of two marker genes of the response to light by Northern blot, showed an exacerbated response in the Δ*env*-1 mutant to both induction and repression of *blu*-1 and *bld*-2, respectively. Furthermore, in a transcriptomic analysis, we found that in the absence of *env*-1, important alterations occur in the blue-light dependent regulation of carbon and amino acid metabolism. Differential expression analysis showed that ENVOY plays an important role in the adjustment of central metabolism in response to light, which is consistent with the growth-deficiency phenotype exhibited by this mutant. Our results show the influence of light receptors on carbon and amino acid metabolism, which is consistent with the findings in *T. reesei* and *N. crassa* (Figure 7; Schmoll et al., 2012; Tisch and Schmoll, 2013). Although in those cases the studies were carried out in liquid medium in shake flasks with cellulose as sole carbon source, and the transcriptomic analysis were carried out after 28 to 72 h of continuous exposure to light, which may have resulted in a strong bias towards the detection of indirect gene targets. Importantly, we observed that *frq*, the orthologue of the *N. crassa* gene encoding Frequency, the master oscillator of the circadian clock, is up-regulated in response to light in a BLR-1 dependent manner, suggesting that as expected light entrainment of the clock in *T. atroviride* depends on the BLR-complex (Cha et al., 2014).

The results described above led us to reason that in *T. atroviride* there is a strong link between ENVOY-dependent early gene regulation and central metabolism. Indeed, when analyzing the growth patterns in different carbon sources in light and dark of the WT strain and the Δ*env*-1 mutant, it was evident that *T. atroviride* utilizes carbon sources differently depending on the light conditions. Furthermore, growth of the *env*-1 mutant was more severely affected when exposed to light, suggesting that ENVOY is a modulator of nutrient signaling during the response to blue light, perhaps modifying the overall metabolic state of cells in the presence and absence of light (Figure 7). In this regard, in *T. reesei*, an interaction between light and nutrient signaling regulated in part by ENVOY has been demonstrated (Schuster et al., 2007; Tisch and Schmoll, 2013; Tisch et al., 2014). These observations show that there are certain mechanisms regulated in the same way in these two *Trichoderma* species despite the contrasting conidiation phenotype observed.

Furthermore, in the transcriptome we found a strong reduction in the number of transcripts responsive to blue light in the Δ*blr*-1 mutant, which shows that it is the main photoreceptor within the blue light perception system in *T. atroviride*. It was recently described in *Fusarium fujikuroi* that a drastic decrease occurs in the mRNA levels of carotenoid genes, as well as morphological and metabolic changes in a mutant of the *wco*A gene, encoding a White-Collar 1 orthologue (Pardo-Medina et al., 2021). This result is quite interesting since this analysis was carried out early (15, 60, and 240 min) upon exposure to constant light of the WT and the wcoA strains and their results coincide with ours at 30 min, even though these two species of fungi are phylogenetically distant.

Our transcriptomics results also showed that *cry*-1 and *cry-DASH* are induced by blue light and are regulated in a BLR-1 dependent manner. Furthermore, we showed that the photoreceptors encoded by these genes do not contribute to the control of mycelial growth. The results of the differential expression analysis showed that there are no major transcriptional changes in the expression profile of the Δ*cry*-1 and Δ*cry-DASH* mutants as compared to the WT in response to light, indicating that there is no significant contribution of CRY-1 and CRY-DASH to the perception of blue light, not at least in the early response to low light intensity (30 min, 200 μmol⋅m^–2^). We found that all genes that are regulated in a CRY-1 and/or Cry-DASH dependent manner are also under the regulation of the BLR-1 photoreceptor, thus, playing a role as secondary elements of the response.

CRY-1, a (6-4)-photolyase, is necessary for photoreactivation in *T. atroviride* and its light induced expression depends on BLR-1 (García-Esquivel et al., 2016). In this sense, our results corroborated the participation of CRY-1 in photoreactivation. The participation of the BLR-1 protein in the regulation of *cry*-1 has also been reported in *T. reesei*, as well as its role in photoreactivation (Guzmán-Moreno et al., 2014). Similarly, in *Ustilago maydis* the Wco1 protein contributes to UV-light tolerance through the regulation of the expression of CPD and 6-4 photolyases (Brych et al., 2016). Likewise, in the entomopathogenic fungus *Metarhizium robertsii*, it has been shown that White-Collar proteins interact to form a light-sensitive transcription factor that regulates the expression of photolyases, necessary to repair damage caused by UV light. The White-Collar proteins are known to even physically interact with photolyases (Peng et al., 2021). These results suggest a conserved role of the White-Collar proteins in the transcriptional regulation of genes that encode proteins with photolyase activity in different fungal lineages.

Our functional category enrichment results clearly showed that ENVOY is a repressor of DNA metabolic processes that are activated by the BLR-1 protein in response to blue light. Interestingly, we found that in the absence of ENVOY there is an evident increase in the levels of the transcripts derived from these genes. In this regard, it has been shown that RAD1 forms a complex with RAD10 in *S. cerevisiae* with the endonuclease activity necessary for nucleotide excision repair (NER), while RAD4 forms a stable complex with RAD23 that specifically binds to UV-light-irradiated DNA (Bailly et al., 1992; Jansen et al., 1998; Boiteux and Jinks-Robertson, 2013). Within the category of DNA metabolic processes, we identified a gene that codes for a RAD23 protein (NER protein), which has been reported in *Beauveria bassiana* to physically interact with the PHR2 photolyase for the repair of DNA lesions by the degradation of (6-4) -pyrimidine-pyrimidine photoproducts (Wang et al., 2020). Within the set of genes with the highest expression in the mutant, we also found *phr*-1, a gene that codes for a photolyase that participates in the repair of pyrimidine cyclobutane dimers caused by UV light in *T. atroviride* (Berrocal-Tito et al., 2007). Based on these observations, we propose a transcriptional co-regulation between ENVOY, the DNA photolyase PHR-1 and RAD proteins during DNA damage repair in *T. atroviride*. Accordingly, we observed a greater germination capacity of Δ*env*-1 conidia than that of the WT after UV-irradiation, demonstrating that ENVOY controls DNA photorepair. These results allow us to propose that ENVOY is the connection between DNA repair processes and metabolic redirecting of resources, since in the mutant of this gene it is evident that there is a greater capacity for DNA repair and an arrest of DNA metabolism (Figure 7). In addition, our data support the Esquivel-Naranjo et al. (2016) discoveries since we also observed that BLR-1 induces the expression of *tmk*-3, indicating that there is a connection between the MAPK stress pathway and photorepair. In this work, we showed that ENVOY also plays a role in the regulation of *tmk*-3 transcription, taking these results into consideration, we suggest that this photoreceptor plays a key role in the crosstalk between the MAPK signaling pathway and photoperception (Figure 7).

It is evident that filamentous fungi when facing sunlight need to control the level of response to this stimulus depending on the dose, since high radiation represents a risk for their genomic integrity, but in low dosages, light constitutes a cue for entry into development. In this work, using the minimum light intensity at which *T. atroviride* responds to produce conidia, we determined the metabolic processes that are finely regulated in response to this stimulus. Moreover, in general, regardless of the type of stress, it is expected that proteins such as ENVOY exist distributed in living organisms that function as a kind of buffer, which establish the threshold of stress responses.

## Data Availability Statement

The datasets presented in this study can be found in online repositories. The names of the repository/repositories and accession number(s) can be found in section “Materials and Methods” and as footnotes.

## Author Contributions

EP-S, PM-H, EB-H, EE-N, and NC-V conducted wet-lab experiments. EP-S, JV-E, and AH-E analyzed data and wrote the manuscript. All authors read and approved the manuscript.

## Conflict of Interest

The authors declare that the research was conducted in the absence of any commercial or financial relationships that could be construed as a potential conflict of interest.

## Publisher’s Note

All claims expressed in this article are solely those of the authors and do not necessarily represent those of their affiliated organizations, or those of the publisher, the editors and the reviewers. Any product that may be evaluated in this article, or claim that may be made by its manufacturer, is not guaranteed or endorsed by the publisher.

## Footnotes

1
https://blast.ncbi.nlm.nih.gov/Blast.cgi?PAGE=Proteins

2
https://www.bioinformatics.babraham.ac.uk/projects/fastqc/

3
http://genome.jgi.doe.gov/Triat2/Triat2.home.html

4
https://www.ncbi.nlm.nih.gov/geo/query/acc.cgi?acc=GSE165935

## Supplementary Material

Supplementary Figure 1 Phylogenetic analysis of ENVOY protein in various filamentous fungi. Phylogenetic relationships of *T. atroviride* ENVOY with representative fungal counterparts. The NCBI access code of each ENVOY or VIVID protein and its sequence identity are presented in parentheses after each fungal name, respectively. Bootstrap values of 1000 are given at the nodes. Scale: length of the branch proportional to the genetic distance evaluated with the neighbor-joining method in MEGA X.

Supplementary Figure 2 General characterization of *env1* gene replacement mutants. Confirmation of gene replacement event. A PCR was carried out using a primer within the coding sequence of the *hph* gene (hygromycin resistance cassette) as forward and a primer derived from the UTR region of the *env*-1 gene as reverse. In this experiment the amplifying strains had integration of the construct in the correct region of the genome **(A).** PCR to amplify the *env*-1 gene. In this experiment the strains that do not show amplification are pure mutants **(B)**. Light induced conidiation of four *env-*1 independent mutants **(C)**.

Supplementary Figure 3 Total growth of colonies under constant darkness. Growth was measured every 12 h for 72 h under darkness on potato dextrose agar (PDA) (*n* = 8).

Supplementary Figure 4 Effects of constant blue light on the growth and conidiation at 2.9 μmol.m^–2^s^–1^. The strains were exposed to a 2.9 μmol⋅m^–2^⋅s^–1^ dose of blue light for 72 h. **(A)** Phenotype of the WT strain and the Δ*env-*1 mutant under constant lighting and darkness. **(B)** Conidia per plate of strain WT and mutant Δ*env-*1 under constant illumination.

Supplementary Figure 5 Effects of constant blue light on the growth and conidiation at 11.6 μmol.m^–2^s^–1^. The strains were exposed to a dose of 2.9 μmol⋅m^–2^⋅s^–1^ blue light for 72 h. **(A)** Phenotype of the WT strain and the Δ*env-*1 mutant under constant lighting and darkness. **(B)** Conidia per plate of strain WT and mutant Δ*env-*1 under constant illumination.

Supplementary Figure 6 Distribution of reads mapping to the *env*-1 gene. The figure shows a 3249 nucleotides genomic region containing the *env*-1 gene. The diagram shows the RNA seq reads mapping to this region when the WT strain (red) or the Δ*env*-1 (blue) were exposed to a pulse of blue light. At the bottom of the figure, we show a diagram of the structure of the *env*-1 gene indicating in light green rectangles exons, black lines introns and in dark green rectangles the 5′ and 3′ untranslated regions. Arrow heads indicate the direction of transcription.

Supplementary Figure 7 Genes regulated by Cry-1 y Cry-DASH. **(A)** Venn diagram showing the number of up-regulated genes shared by the WT strain and the Δ*cry*-1 and Δ*cry-DASH* mutants or that are unique for each strain. **(B)** Venn diagram showing the number of down-regulated genes shared by the WT strain and the Δ*cry*-1 and Δ*cry-DASH* mutants or that are unique for each strain.

Supplementary Figure 8 *Trichoderma atroviride* tolerance to UV light. Colonies of the WT strain and the Δ*cry*-1 and Δ*env*-1 mutants were incubated for 48 h in the dark after irradiation with 350 J. m^–2^ UV-C light.
