# Supplementary figures and images for: A Global Analysis of Photoreceptor-Mediated Transcriptional Changes Reveals the Intricate Relationship Between Central Metabolism and DNA Repair in the Filamentous Fungus Trichoderma atroviride
Source: Front Microbiol. 2021 Sep 8;12:724676. doi: 10.3389/fmicb.2021.724676 (PMC8456097; doi:10.3389/fmicb.2021.724676)

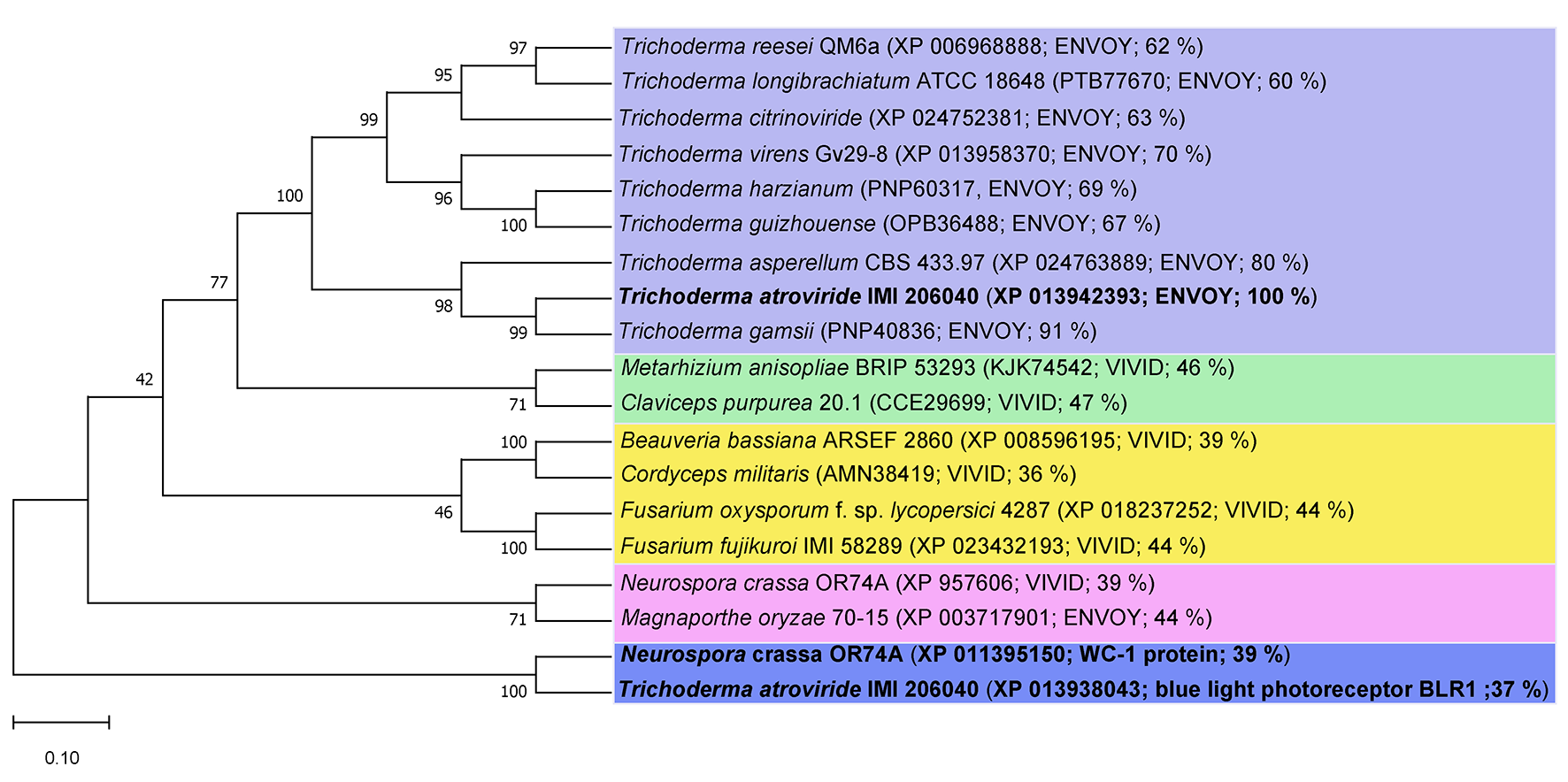

Supplement: Supplementary Figure 1 — Phylogenetic analysis of ENVOY protein in various filamentous fungi. Phylogenetic relationships of T. atroviride ENVOY with representative fungal counterparts. The NCBI access code of each ENVOY or VIVID protein and its sequence identity are presented in parentheses after each fungal name, respectively. Bootstrap values of 1000 are given at the nodes. Scale: length of the branch proportional to the genetic distance evaluated with the neighbor-joining method in MEGA X. [file Image_1.TIFF]

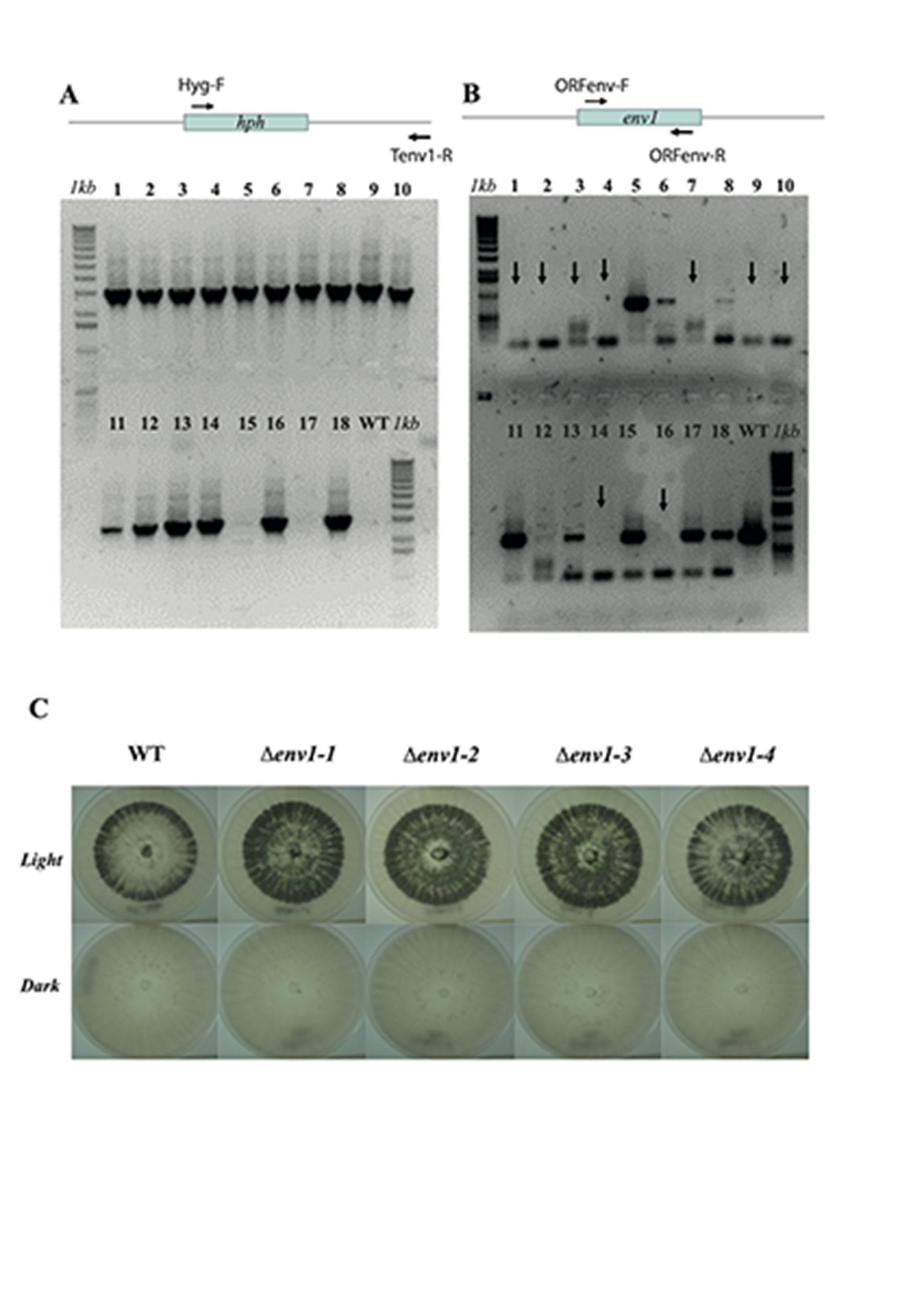

Supplement: Supplementary Figure 2 — General characterization of env1 gene replacement mutants. Confirmation of gene replacement event. A PCR was carried out using a primer within the coding sequence of the hph gene (hygromycin resistance cassette) as forward and a primer derived from the UTR region of the env-1 gene as reverse. In this experiment the amplifying strains had integration of the construct in the correct region of the genome (A). PCR to amplify the env-1 gene. In this experiment the strains that do not show amplification are pure mutants (B). Light induced conidiation of four env-1 independent mutants (C). [file Image_2.TIF]

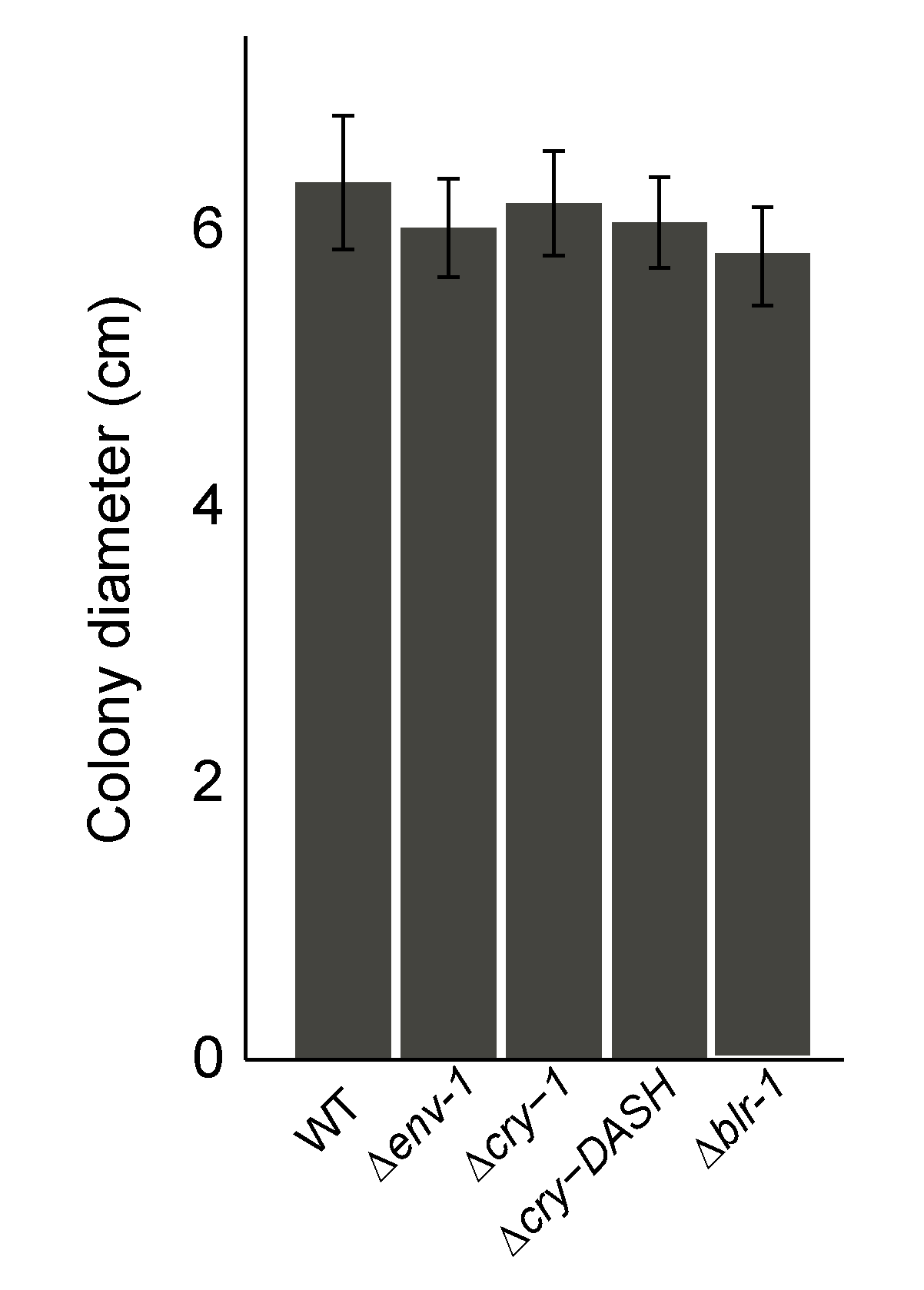

Supplement: Supplementary Figure 3 — Total growth of colonies under constant darkness. Growth was measured every 12 h for 72 h under darkness on potato dextrose agar (PDA) (n = 8). [file Image_3.TIFF]

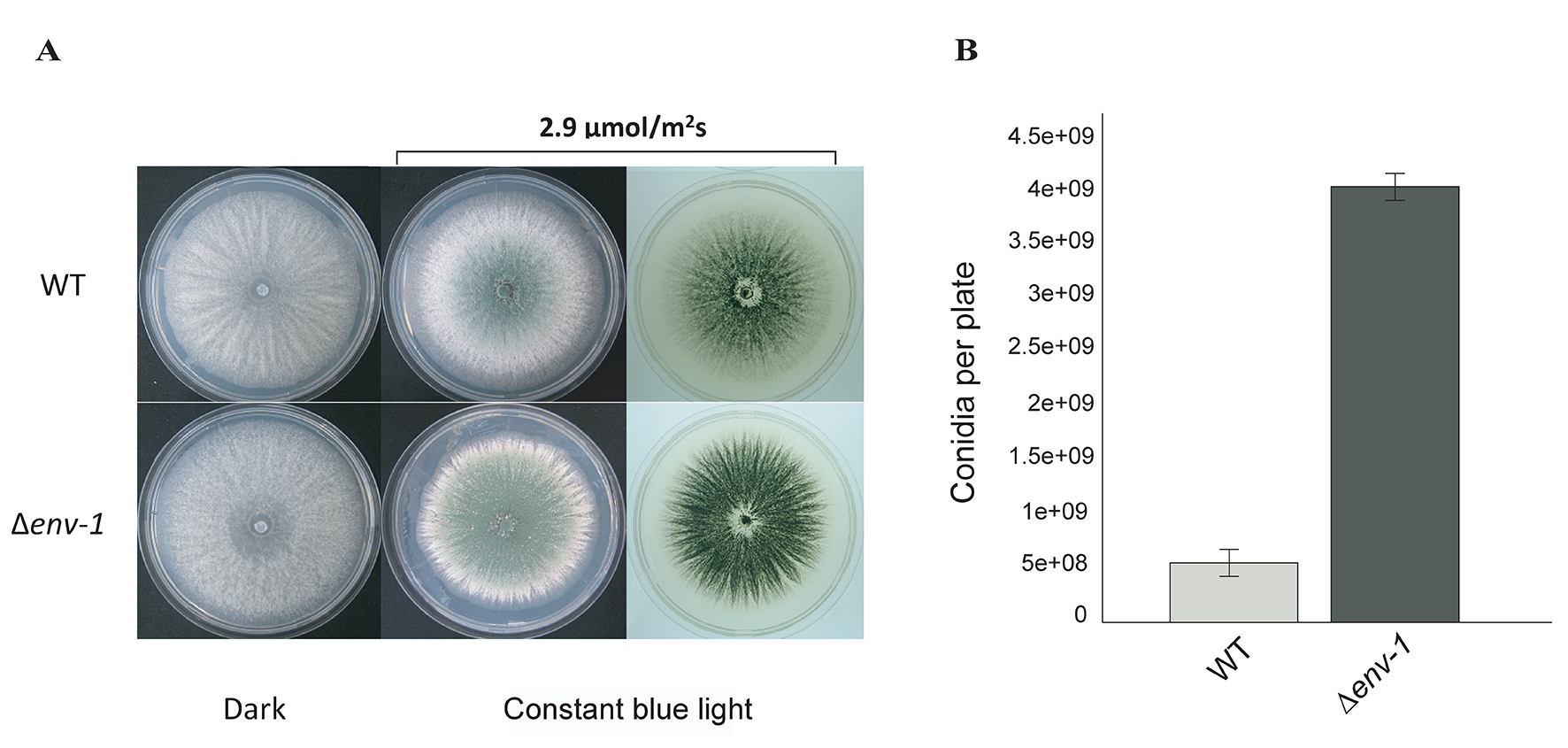

Supplement: Supplementary Figure 4 — Effects of constant blue light on the growth and conidiation at 2.9 μmol.m–2s–1. The strains were exposed to a 2.9 μmol⋅m–2⋅s–1 dose of blue light for 72 h. (A) Phenotype of the WT strain and the Δenv-1 mutant under constant lighting and darkness. (B) Conidia per plate of strain WT and mutant Δenv-1 under constant illumination. [file Image_4.TIFF]

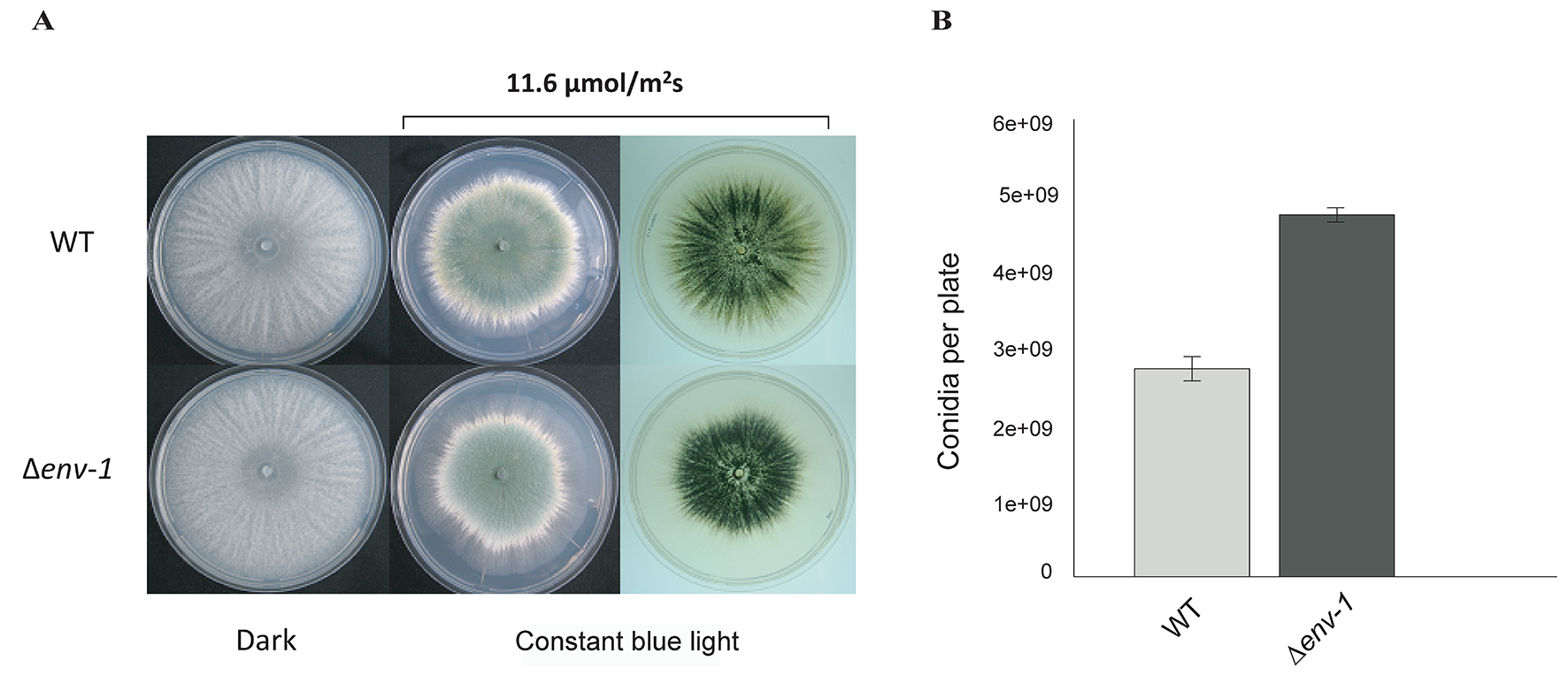

Supplement: Supplementary Figure 5 — Effects of constant blue light on the growth and conidiation at 11.6 μmol.m–2s–1. The strains were exposed to a dose of 2.9 μmol⋅m–2⋅s–1 blue light for 72 h. (A) Phenotype of the WT strain and the Δenv-1 mutant under constant lighting and darkness. (B) Conidia per plate of strain WT and mutant Δenv-1 under constant illumination. [file Image_5.TIFF]

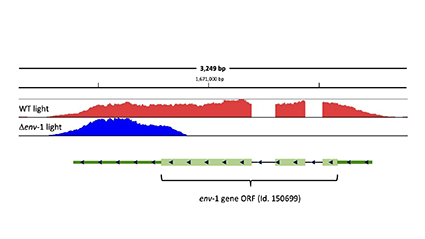

Supplement: Supplementary Figure 6 — Distribution of reads mapping to the env-1 gene. The figure shows a 3249 nucleotides genomic region containing the env-1 gene. The diagram shows the RNA seq reads mapping to this region when the WT strain (red) or the Δenv-1 (blue) were exposed to a pulse of blue light. At the bottom of the figure, we show a diagram of the structure of the env-1 gene indicating in light green rectangles exons, black lines introns and in dark green rectangles the 5′ and 3′ untranslated regions. Arrow heads indicate the direction of transcription. [file Image_6.TIFF]

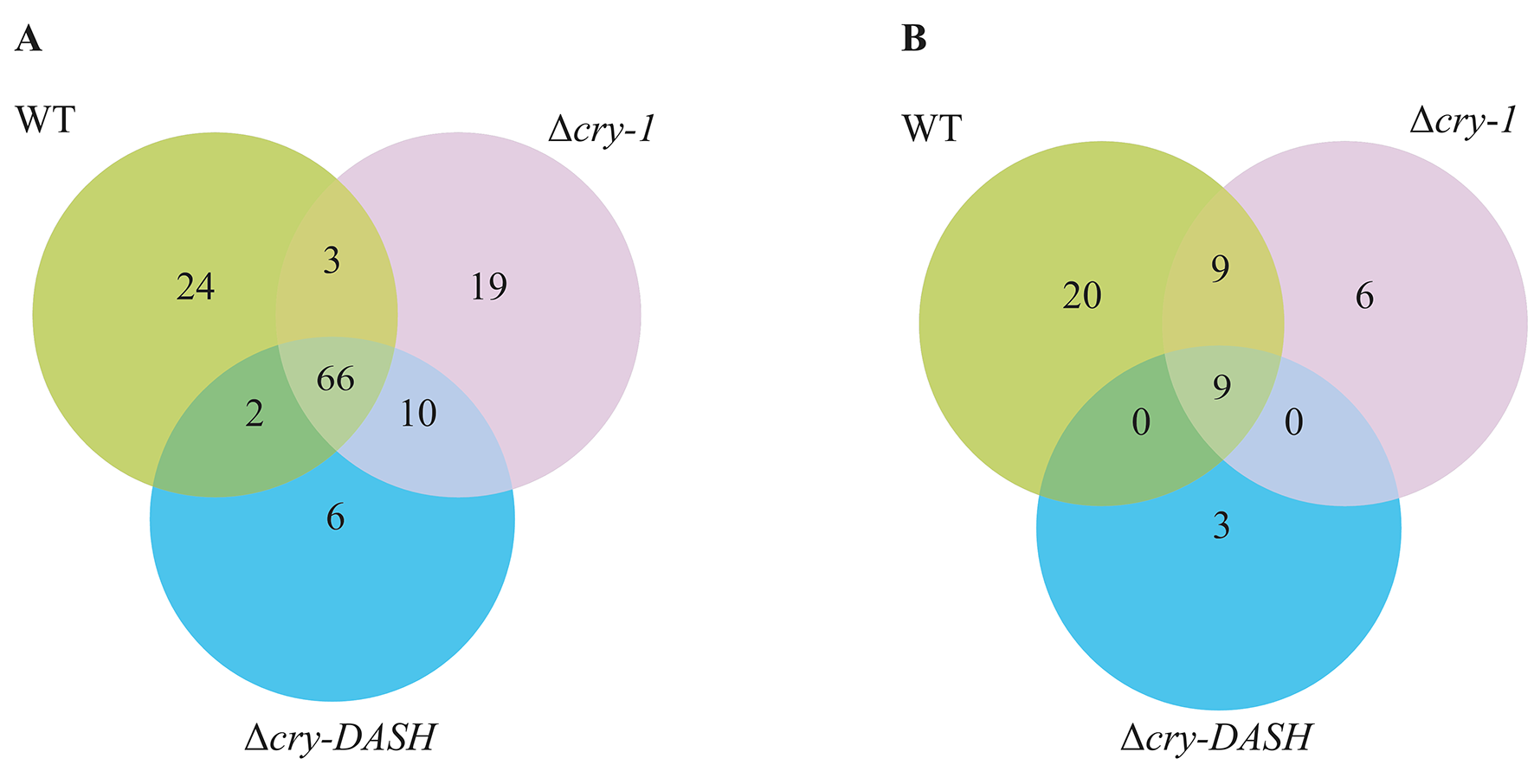

Supplement: Supplementary Figure 7 — Genes regulated by Cry-1 y Cry-DASH. (A) Venn diagram showing the number of up-regulated genes shared by the WT strain and the Δcry-1 and Δcry-DASH mutants or that are unique for each strain. (B) Venn diagram showing the number of down-regulated genes shared by the WT strain and the Δcry-1 and Δcry-DASH mutants or that are unique for each strain. [file Image_7.TIFF]

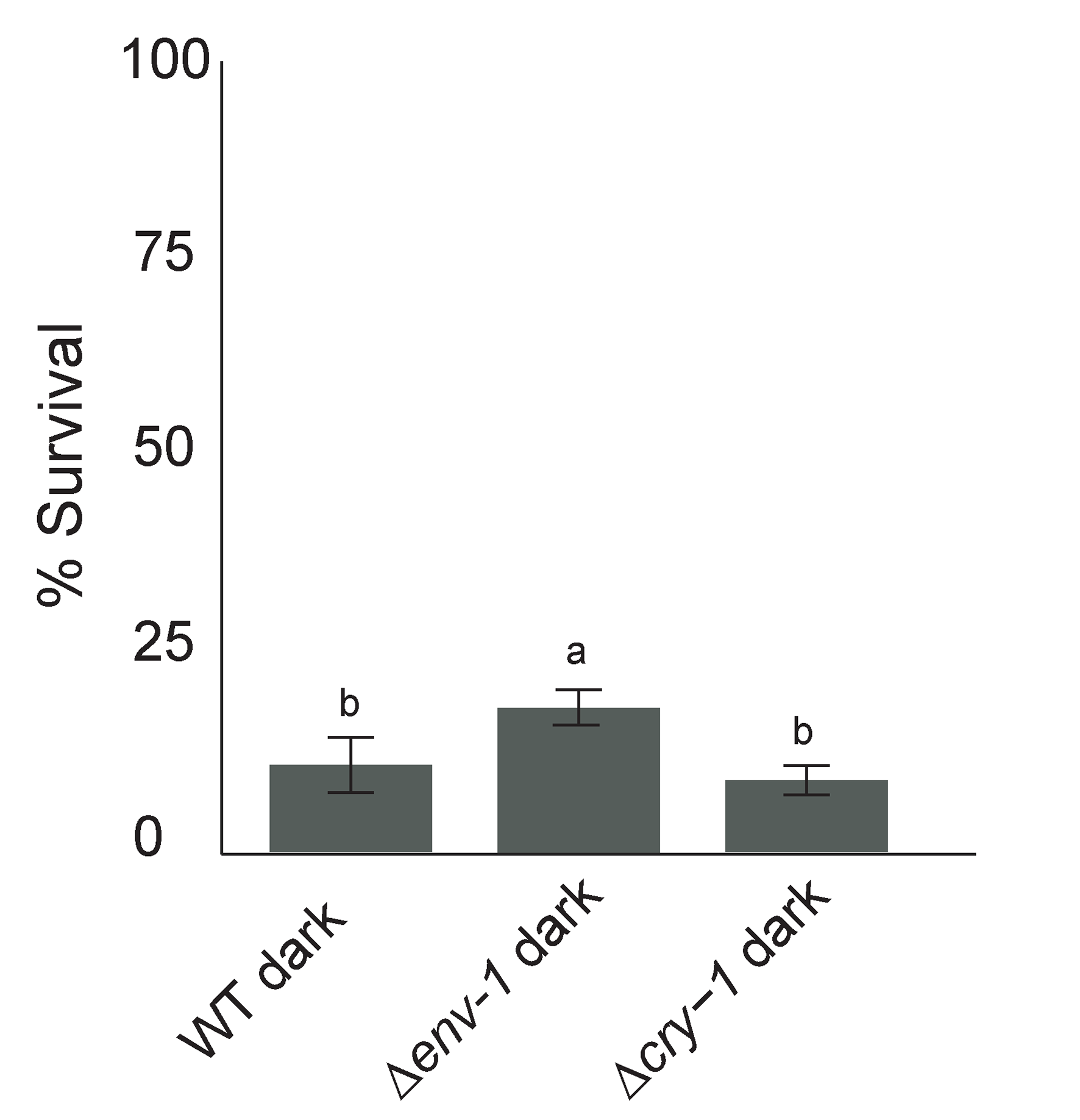

Supplement: Supplementary Figure 8 — Trichoderma atroviride tolerance to UV light. Colonies of the WT strain and the Δcry-1 and Δenv-1 mutants were incubated for 48 h in the dark after irradiation with 350 J. m–2 UV-C light. [file Image_8.TIFF]
