# Supplementary material for: A Global Analysis of Photoreceptor-Mediated Transcriptional Changes Reveals the Intricate Relationship Between Central Metabolism and DNA Repair in the Filamentous Fungus Trichoderma atroviride
Source: Front Microbiol. 2021 Sep 8;12:724676. doi: 10.3389/fmicb.2021.724676 (PMC8456097; doi:10.3389/fmicb.2021.724676)
Supplement: Supplementary file 10 [file Data_Sheet_2.docx]

**Supplementary figures**

**Figure S1. Phylogenetic analysis of ENVOY protein in various filamentous fungi.** Phylogenetic relationships of *T. atroviride* ENVOY with representative fungal counterparts. The NCBI access code of each ENVOY or VIVID protein and its sequence identity are presented in parentheses after each fungal name, respectively. Bootstrap values of 1000 are given at the nodes. Scale: length of the branch proportional to the genetic distance evaluated with the neighbor-joining method in MEGA X.

**Figure S2. General characterization of *env1* gene replacement mutants*.*** Confirmation of gene replacement event. A PCR was carried out using a primer within the coding sequence of the *hph* gene (hygromycin resistance cassette) as forward and a primer derived from the UTR region of the *env*-1 gene as reverse. In this experiment the amplifying strains had integration of the construct in the correct region of the genome **(a).** PCR to amplify the *env*-1 gene. In this experiment the strains that do not show amplification are pure mutants (**b**). Light induced conidiation of four *env-*1 independent mutants (**c**).

**Figure S3. Total growth of colonies under constant darkness**. Growth was measured every 12 hours for 72 hours under darkness on potato dextrose agar (PDA) (n = 8).

**Figure S4. Effects of constant blue light on the growth and conidiation at 2.9 μmol.m^-2^s^-1^.** The strains were exposed to a 2.9 μmol·m^-2^·s^-1^ dose of blue light for 72 hours. **(a)** Phenotype of the WT strain and the Δ*env-*1 mutant under constant lighting and darkness. **(b)** Conidia per plate of strain WT and mutant Δ*env-*1 under constant illumination.

**Figure S5. Effects of constant blue light on the growth and conidiation at 11.6 μmol.m^-2^s^-1^.** The strains were exposed to a dose of 2.9 μmol·m^-2^·s^-1^ blue light for 72 hours. **(a)** Phenotype of the WT strain and the Δ*env-*1 mutant under constant lighting and darkness. **(b)** Conidia per plate of strain WT and mutant Δ*env-*1 under constant illumination.

**Figure S6. Distribution of reads mapping to the *env*-1 gene.** The figure shows a 3249 nucleotides genomic region containing the env-1 gene. The diagram shows the RNA seq reads mapping to this region when the WT strain (red) or the Δ*env*-1 (blue) were exposed to a pulse of blue light. At the bottom of the figure, we show a diagram of the structure of the *env*-1 gene indicating in light green rectangles exons, black lines introns and in dark green rectangles the 5’ and 3’ untranslated regions. Arrow heads indicate the direction of transcription.

**Figure S7. Genes regulated by Cry-1 y Cry-DASH. (a)** Venn diagram showing the number of up-regulated genes shared by the WT strain and the Δ*cry-1* and Δ*cry-DASH* mutants or that are unique for each strain. **(b)** Venn diagram showing the number of down-regulated genes shared by the WT strain and the Δ*cry-1* and Δ*cry-DASH* mutants or that are unique for each strain.

**Figure S8. *Trichoderma atroviride* tolerance to UV light.** Colonies of the WT strain and the Δ*cry-1* and Δ*env-1* mutants were incubated for 48 hours in the dark after irradiation with 350 J. m^-2^ UV-C light.
